# Antigen-driven EGR2 expression is required for exhausted CD8^+^ T cell stability and maintenance

**DOI:** 10.1038/s41467-021-23044-9

**Published:** 2021-05-13

**Authors:** Mayura V. Wagle, Stephin J. Vervoort, Madison J. Kelly, Willem Van Der Byl, Timothy J. Peters, Ben P. Martin, Luciano G. Martelotto, Simone Nüssing, Kelly M. Ramsbottom, James R. Torpy, Deborah Knight, Sinead Reading, Kevin Thia, Lisa A. Miosge, Debbie R. Howard, Renee Gloury, Sarah S. Gabriel, Daniel T. Utzschneider, Jane Oliaro, Jonathan D. Powell, Fabio Luciani, Joseph A. Trapani, Ricky W. Johnstone, Axel Kallies, Christopher C. Goodnow, Ian A. Parish

**Affiliations:** 1 John Curtin School of Medical Research, Australian National University, ACT, Australia; 2 Peter MacCallum Cancer Centre, Melbourne, VIC Australia; 3 Sir Peter MacCallum Department of Oncology, University of Melbourne, Melbourne, VIC Australia; 4 The Kirby Institute for Infection and Immunity, UNSW, Sydney, NSW Australia; 5 Faculty of Medicine, University of New South Wales, Sydney, NSW Australia; 6 Garvan Institute of Medical Research, Darlinghurst, NSW Australia; 7 University of New South Wales, Sydney, NSW Australia; 8 Centre for Cancer Research, VCCC, University of Melbourne, Melbourne, VIC Australia; 9 The Peter Doherty Institute for Infection and Immunity, The University of Melbourne, Melbourne, VIC Australia; 10 Department of Microbiology and Immunology, The University of Melbourne, Melbourne, VIC Australia; 11 The Walter and Eliza Hall Institute of Medical Research, Parkville, VIC Australia; 12 Department of Immunology, Central Clinical School, Monash University, Melbourne, VIC Australia; 13 Bloomberg~Kimmel Institute for Cancer Immunotherapy, Johns Hopkins University of Medicine, Baltimore, MD USA

**Keywords:** Infection, Lymphocytes, CD8-positive T cells, Tumour immunology

## Abstract

Chronic stimulation of CD8^+^ T cells triggers exhaustion, a distinct differentiation state with diminished effector function. Exhausted cells exist in multiple differentiation states, from stem-like progenitors that are the key mediators of the response to checkpoint blockade, through to terminally exhausted cells. Due to its clinical relevance, there is substantial interest in defining the pathways that control differentiation and maintenance of these subsets. Here, we show that chronic antigen induces the anergy-associated transcription factor EGR2 selectively within progenitor exhausted cells in both chronic LCMV and tumours. EGR2 enables terminal exhaustion and stabilizes the exhausted transcriptional state by both direct EGR2-dependent control of key exhaustion-associated genes, and indirect maintenance of the exhausted epigenetic state. We show that EGR2 is a regulator of exhaustion that epigenetically and transcriptionally maintains the differentiation competency of progenitor exhausted cells.

## Abstract (web summary)

Exhausted T cells arise when chronic activation triggers functional defects. Here the authors show that chronic antigenic stimulation in both tumour and infection models induces the expression of EGR2, which drives and stabilises exhausted cell epigenetic and transcriptional identity.

## Introduction

Persistent antigen encounter by CD8^+^ T cells during either chronic viral infection or tumour growth leads to a progressive loss of inflammatory cytokine production and upregulation of inhibitory receptors. This differentiation state is termed T cell exhaustion, and it is transcriptionally and epigenetically distinct from functional effector and memory differentiation^1–5^. While exhaustion likely evolved to limit immunopathology, sustained exhaustion blunts the immune response, thereby contributing to both tumour growth and persistence of chronic infections^6^. For this reason, exhausted cells have emerged as a major target for immunotherapy approaches. In particular, inhibitory receptor blockade, and the concurrent reinvigoration of the exhausted T cell response, have demonstrated striking clinical efficacy in the treatment of cancer^6^.

Recent findings on the dynamics of T cell differentiation during exhaustion have greatly advanced our understanding of both exhausted cell maintenance and the response to checkpoint blockade. After commitment to the exhausted state, T cells enter an equilibrium whereby stem-like “progenitor” exhausted cells constitutively and progressively differentiate into more proliferative, effector-like “transitory” exhausted cells, before entering a non-proliferative, terminally exhausted state^7–20^. Importantly, the progenitor exhausted cell population (identified by TCF1 and Slamf6 expression) is the primary exhausted T cell population that both sustains the response in the steady-state, and expands upon checkpoint blockade^9,11,13,15,17,18^, and cancer patients lacking this TCF1^+^ subset are unresponsive to therapy^21^. Thus, a better understanding of the pathways that regulate progenitor CD8^+^ T cell differentiation and function during exhaustion is critical to the development of more efficacious cancer immunotherapy approaches.

There are parallels between the transcriptional networks engaged in CD8^+^ T cell exhaustion and CD4^+^ T cell anergy. In particular, antigen-induced NFAT-dependent regulatory programs, including NFAT-dependent induction of NR4A factors, regulate both CD4^+^ T cell anergy and CD8^+^ T cell exhaustion^22–24^. EGR2 is a transcriptional master regulator of anergy that is also induced downstream of TCR-induced NFAT activation^24,25^. EGR2 inhibits T cell function during anergy through direct induction of factors that diminish TCR signalling, such as *Cblb*, *Dgka* and *Dgkz*^26,27^, and *Egr2*^−/−^ T cells are resistant to anergy induction^25,26^. We observed that *Egr2* expression was elevated in exhausted CD8^+^ T cells within published datasets^1,3^ suggesting that EGR2 may also regulate exhaustion.

Here, we show that persistent antigen recognition drives higher EGR2 expression within exhausted versus effector CD8^+^ T cells. EGR2 was selectively expressed within the TCF1^+^ progenitor exhausted cells and lost upon commitment to more differentiated states. Moreover, EGR2 played an indispensable role in the regulation of progenitor exhausted cell differentiation through both transcriptional and epigenetic control of the exhausted state, although not via the same gene targets as in in vitro CD4^+^ T cell anergy. Collectively, these data demonstrate that EGR2 is a key transcriptional and epigenetic regulator of the exhausted state.

## Results

### EGR2 is selectively expressed in TCF1^+^ progenitor exhausted T cells

**Fig. 1:**
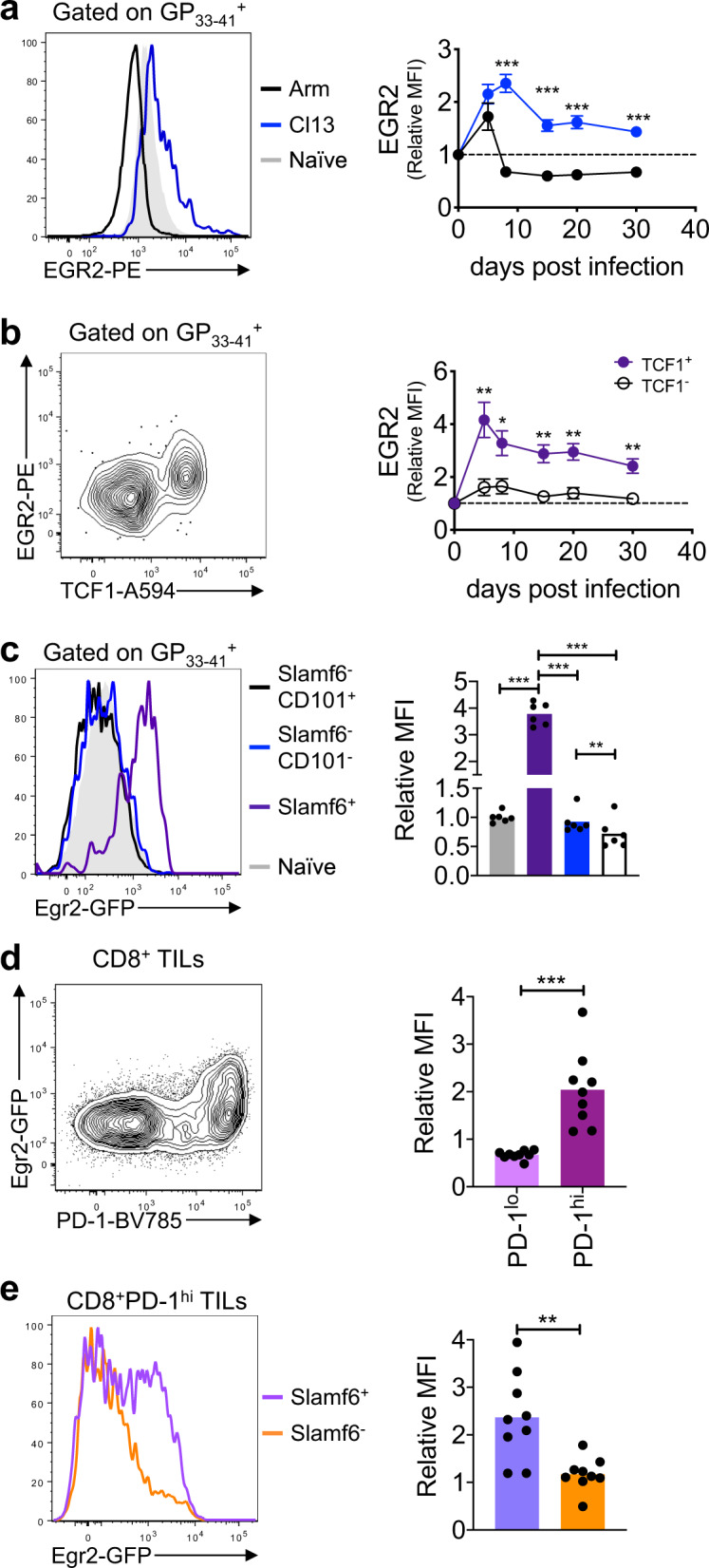
EGR2 expression is elevated within TCF1^+^ progenitor exhausted cells. **a** EGR2 protein levels assessed by flow cytometry within splenic H2-D^b^GP_33–41_ tetramer-stained CD8^+^ T cells from B6 mice infected with LCMV-Arm or Cl13 for the indicated times. Representative histogram (left) shows data from day 8 p.i., with naive T cell staining included from polyclonal CD44^lo^CD8^+^ T cells from uninfected B6 mice. Right graph shows EGR2 expression relative to naive levels (as defined above). *n* = 11–15 mice per group per time point from 2–3 independent experiments. p-values were calculated using a two-tailed unpaired *T* test (days 5, 15, 20) or two-tailed Mann–Whitney test (days 8, 30). For all indicated significant differences *p* < 0.0001. **b** EGR2 protein levels assessed as in **a** within TCF1^+^ and TCF1^-^ tetramer^-^stained CD8^+^ T cells from B6 mice infected with LCMV-Cl13 for the indicated times. Representative plot (left) shows data from day 20 p.i. Right graph shows EGR2 expression relative to naive levels (as defined above). *n* = 5–6 mice per group per time point from two independent experiments. *p*-values were calculated using a two-tailed unpaired *T* test. For indicated significant differences, exact *p* values (left to right) = 0.0060, 0.0141, 0.0035, 0.0022, 0.0024. **c** Egr2-GFP reporter expression within polyclonal CD44^lo^CD8^+^ T cells, or Slamf6^+^, Slamf6^-^CD101^-^ and Slamf6^-^CD101^+^ splenic H2^-^D^b^GP_33-41_ tetramer-stained CD8^+^ T cells from chronic LCMV infected Egr2-GFP reporter mice at day 20 p.i. Representative histogram (top) and pooled data (bottom) are shown with *n* = 6 mice from two independent experiments. *p*-values were calculated using a one-way ANOVA with a Tukey’s post-test. For indicated significant differences, exact *p* values (left to right) = <0.0001, 0.0002, 0.0002, 0.0043. **d**, **e** Egr2-GFP mice were injected s.c. with 2 × 10^5^ B16-OVA cells, and at day 14 post-injection TILs were isolated from tumours for analysis. GFP levels in PD-1^hi^ vs PD-1^lo^ total CD45^+^CD8^+^ TILs (**d**), and Slamf6^+^ vs Slamf6^-^ CD45^+^CD8^+^PD-1^hi^ TILs (**e**) are shown. Representative plots/histograms are on the left, and pooled data are on the right showing MFI relative to naive. *n* = 9 mice per group from two independent experiments. *p*-values were calculated using a two-tailed Mann–Whitney test, exact *p* values =  <0.0001 (**d**), 0.0028 (**e**). Error bars depict SEM, *p < 0.05, **p < 0.01, ***p < 0.001.

During mouse infection with chronic lymphocytic choriomeningitis virus (LCMV) Clone 13 (Cl13), virus-specific CD8^+^ T cells adopt an exhausted state and fail to clear the infection. In contrast, antigen-specific CD8^+^ T cells during acute LCMV Armstrong (Arm) infection form polyfunctional effector cells that contribute to the clearance of infection and differentiate into memory cells. To directly measure EGR2 expression in chronic and acute LCMV infection, we examined EGR2 protein levels by flow cytometry within tetramer-stained H-2D^b^GP_33-41_-specific CD8^+^ T cells (GP33^+^ cells, Supplementary Fig. 1a) isolated from the spleens of C57BL/6 (B6) mice infected with either LCMV-Cl13 or Arm. During acute LCMV-Arm infection, EGR2 expression initially spiked above naive CD8^+^ T cell levels at day 5 post-infection (p.i.) before dropping below naive levels after viral clearance from day 8 p.i. onwards (Fig. 1a). In contrast, during chronic LCMV-Cl13 infection, EGR2 was significantly higher than in LCMV-Arm infection from day 8 p.i. onwards, and remained elevated in antigen-specific CD8^+^ T cells throughout infection. Thus, EGR2 expression is elevated in antigen-specific CD8^+^ T cells in chronic relative to acute LCMV infection.

We next examined the dynamics of EGR2 expression during the exhaustion differentiation process. Notably, among antigen-specific CD8^+^ T cells, EGR2 protein expression was limited to the TCF1^+^ progenitor population at all time-points examined (Fig. 1b). To map *Egr2* expression independently and in more detail, we examined *Egr2* expression within antigen-specific T cells using GFP knock-in *Egr2* reporter (Egr2-GFP) mice^28^. We observed elevated Egr2-GFP reporter expression selectively within progenitor exhausted (Slamf6^+^) cells (Fig. 1c), while it was low in all other sub-populations, including transitory (CD101^−^) or terminally (CD101^+^) exhausted cells^10^ (Fig. 1c). Similar results were seen within CD8^+^ tumour infiltrating lymphocytes (TILs; Supplementary Fig. 1e) isolated from sub-cutaneous (s.c.) B16-OVA transgenic tumours 14 days after inoculation. Egr2-GFP expression was restricted to PD-1^hi^ cells (Fig. 1d) and selectively elevated within Slamf6^+^ progenitor cells (Fig. 1e). Thus, EGR2 expression is enriched within progenitor exhausted cells.

Notably, we consistently observed an EGR2 negative sub-population of progenitor cells (28.8% ± 5.3% S.D. of progenitor cells) (Fig. 1c). To better understand the difference between EGR2^+^ and EGR2^−^ progenitor exhausted cells, we conducted RNAseq gene expression profiling of these subsets (Supplementary Fig. 1b). GFP^+^ cells expressed higher levels of progenitor subset-specific genes, while GFP^−^ cells had higher expression of genes associated with a more differentiated state (Supplementary Fig. 1c, Supplementary Data 1). Recent data has shown that progenitor exhausted cells reversibly switch between two subsets: a more quiescent CD69^+^ subset, and a more proliferative CD69^−^ subset en route to differentiation^7^. Strikingly, there was a very strong enrichment of the CD69^+^ progenitor signature within GFP^+^ cells, and an equally strong enrichment of the CD69^−^ signature within GFP^-^ cells, as assessed by Gene Set Enrichment Analysis (GSEA) (Supplementary Fig. 1d). Thus, EGR2 positive cells represent a less differentiated progenitor sub-population.

### EGR2 expression is maintained by chronic antigen encounter

**Fig. 2:**
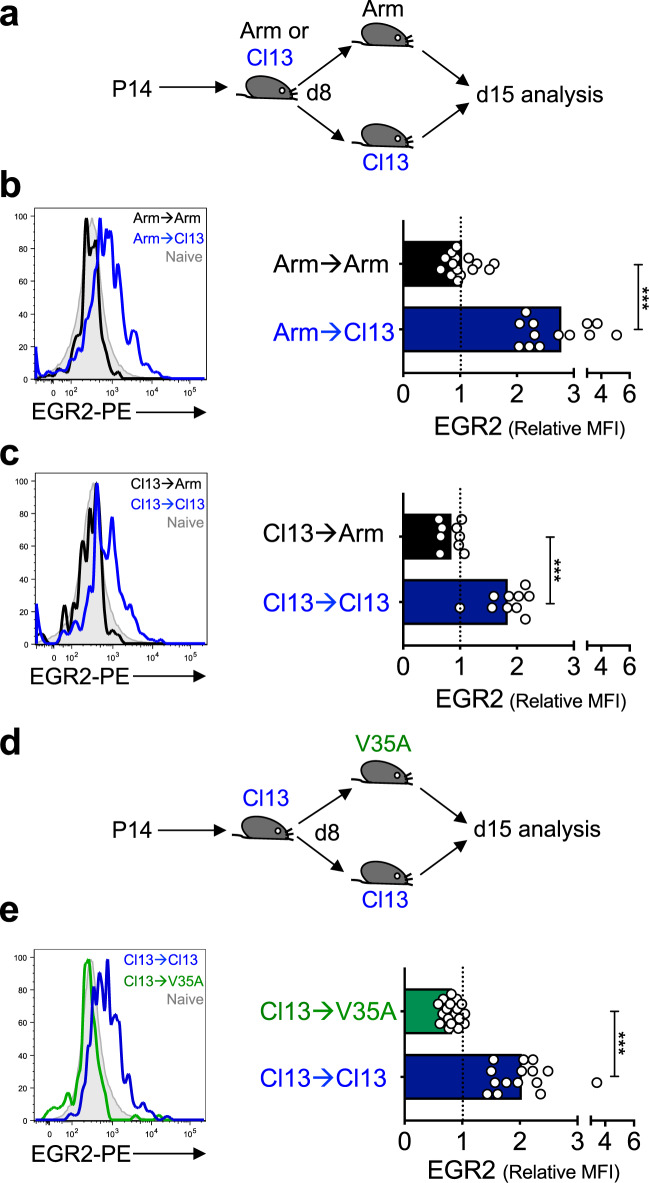
EGR2 expression is maintained by chronic antigen. **a** Experiment outline for **b**, **c**. 2 × 10^3^ CD45.1^+^ P14 cells were transferred into B6 mice subsequently infected with LCMV-Arm or LCMV-Cl13. On day 8 p.i., splenocytes were isolated and the equivalent of 5 × 10^5^ P14 cells were transferred into LCMV-Cl13 or LCMV-Arm infection-matched B6 mice. Seven days later (day 15 p.i.), EGR2 protein levels were assessed within the transferred P14 cells. **b**, **c** EGR2 levels within cells transferred from LCMV-Arm (**b**) or LCMV-Cl13 (**c**) donors into Arm or Cl13 infected recipients. Left histograms show representative plots, with naive CD44^lo^PD-1^lo^ cells from Cl13 infected mice also shown in grey. Right graphs show EGR2 MFI relative to the above naive population. *n* = 13 (top) and 15 (bottom) (**b**), and *n* = 10 (top) and 9 (bottom) (**c**), mice per group from 2–3 independent experiments. **d** Experiment outline for **e**. Experiment was conducted as in **a** except that P14 cells derived from Cl13 infected donors were transferred into recipients infected with wild-type Cl13 or GP V35A mutant Cl13. **e** EGR2 levels within cells transferred from Cl13 donors into Cl13 or V35A recipients. Plots and data representation is as in **b**, **c**. *n* = 15 (top) and 18 (bottom) mice per group from three independent experiments. *p*-values were calculated using a two-tailed unpaired *T* test (**c**) or two-tailed Mann–Whitney test (**b**, **e**). For all indicated significant differences *p* < 0.0001. ***p < 0.001.

During both acute and chronic LCMV infection, EGR2 levels correlated with viral load (Fig. 1a). Coupled with the fact that EGR2 is directly induced downstream of TCR signalling via NFAT during anergy induction^24^, we thus speculated that EGR2 expression is maintained within exhausted CD8^+^ T cells by chronic antigen encounter. To test this idea, 2 × 10^3^ CD45.1^+^ H-2D^b^GP_33–41_-specific CD8^+^ TCR transgenic (P14) T cells were transferred into B6 mice that were subsequently infected with LCMV-Arm. On day 8 p.i., 5 × 10^5^ splenic P14 cells were isolated from these mice and transferred into either LCMV-Arm or LCMV-Cl13 B6 recipients that were also infected 8 days previously. At this time-point, virus is present at high titres in LCMV-Cl13 infected mice, while it is cleared in LCMV-Arm infected mice, meaning that the P14 cells were transferred into recipients that did or did not contain virus. 7 days later (day 15 p.i.), EGR2 expression was assessed within the transferred P14 cells (Fig. 2a). Transfer of P14 cells from LCMV-Arm infection into LCMV-Cl13 infected hosts led to increased EGR2 expression relative to P14 cells transferred into control LCMV-Arm infected mice (Fig. 2b). In the reciprocal experiment, the transfer of P14 cells from LCMV-Cl13 infection into LCMV-Arm infected hosts caused loss of EGR2 expression relative to cells transferred into control LCMV-Cl13 infected mice (Fig. 2c). Thus, active infection maintains EGR2 expression.

Active infection could maintain EGR2 via antigen or inflammatory signals. To discriminate between these possibilities, P14 cells derived from LCMV-Cl13 infection were transferred into infection-matched recipients given either LCMV-Cl13, or a mutant form of Cl13 bearing a single point mutation (V35A) within the GP_33-41_ epitope that abolishes peptide binding to H-2D^b^, thereby depriving P14 cells of antigen^29^ (Fig. 2d). Transfer of P14 cells into V35A infected recipients led to loss of EGR2 expression relative to cells transferred into Cl13 infected control mice, despite persistent infection in both hosts^30^ (Fig. 2e). Thus, persistent antigen engagement is required to sustain EGR2 expression during chronic LCMV infection.

### EGR2 regulates inhibitory receptor expression

**Fig. 3:**
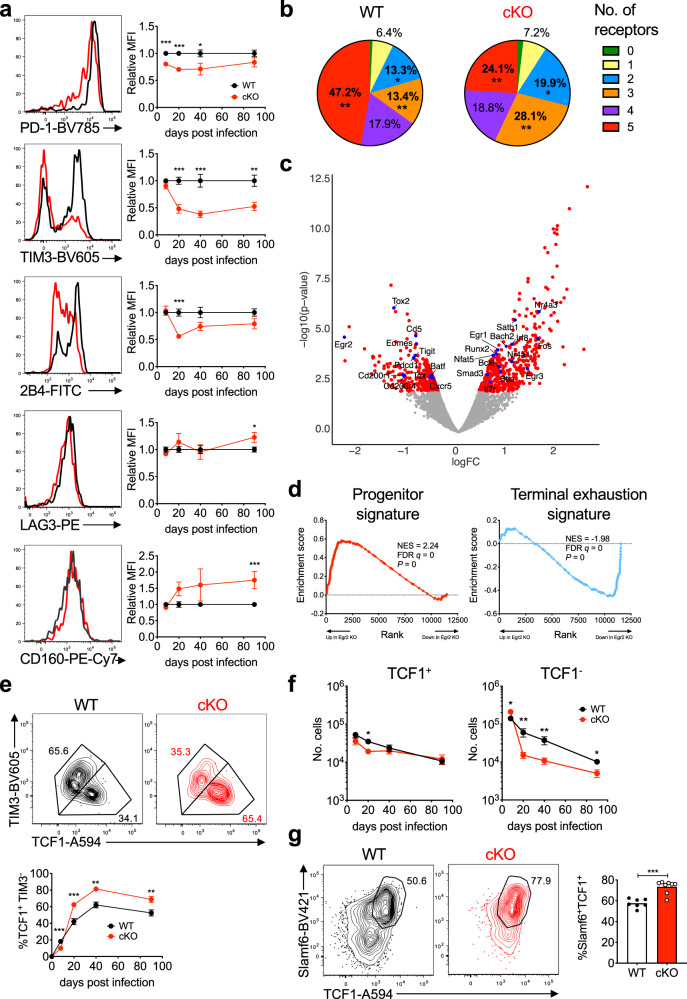
EGR2 maintains the exhausted CD8^+^ T cell response. **a**–**f** WT or cKO mice were depleted of CD4^+^ T cells then infected with LCMV-Cl13 and the response tracked over time. **a** Inhibitory receptor MFI within H2-D^b^GP_33-41_ tetramer-stained CD8^+^ T cells over time (right; normalized to WT mean at each time-point) with representative day 20 p.i. histograms (left). *p*-values were calculated using a two-tailed Mann–Whitney test (2B4 day 20, CD160 day 90) or a two-tailed unpaired *T* test (all others). For indicated significant differences, exact *p* values (left to right) for PD-1= <0.0001, <0.0001, 0.0388, TIM3= <0.0001, 0.0005, 0.0044, 2B4= <0.0001, LAG3 = 0.0247, CD160 = 0.0004. From left to right in the graphs, WT *n* = 12, 10, 7, 13, and cKO *n* = 12, 10, 7, 8, from two independent experiments. **b** Summary of data in **a** expressed as the proportion of cells expressing 0–5 of the listed inhibitory receptors at day 20 p.i. *p*-values were calculated using a two-tailed unpaired *T* test comparing WT to cKO values in each quadrant. For indicated significant differences, exact *p* values = 0.0028 (red), 0.0029 (orange), 0.0212 (blue). **c**–**d** H2-D^b^GP_33–41_ tetramer-stained CD8^+^ T cells were sorted at day 20 p.i. and subjected to RNAseq analysis (*n* = 2 per genotype). **c** Volcano plot denoting differentially expressed genes in cKO versus WT cells. **d** GSEA analysis in cKO vs WT cells of genes selectively up or down within progenitor vs terminally exhausted virus-specific CD8^+^ T cells isolated from chronic LCMV infection. Plots show enrichment of progenitor (left; red) or terminal exhaustion (right; blue) gene signatures. Enrichment score *p*-values were calculated by the GSEA program using an empirical phenotype-based permutation test procedure. **e** Representative (top; day 20 p.i.) and pooled (bottom) proportions of tetramer+ cells that were TIM3^-^TCF1^+^ over the course of infection from the experiment in **a**. *p*-values were calculated using a two-tailed Mann–Whitney test (day 8, 40) or a two-tailed unpaired *T* test (all others). For indicated significant differences, exact *p* values (left to right)= <0.0001, 0.0003, 0.0041, 0.0087. **f** Numbers of TCF1^+^ and TCF1^-^ cells from the experiment in **e**. *p*-values were calculated using a two-tailed Mann–Whitney test (TCF1^-^ day 20, 40) or a two-tailed unpaired *T* test (all others). For indicated significant differences, exact p values (left to right) for TCF1^+^ = 0.0101, for TCF1^-^ = 0.0156, 0.0015, 0.0059, 0.0317. **g** %Slamf6^+^TCF1^+^ cells of PD-1^hi^CD8^+^ TILs in WT vs CD8 cKO mice given B16-OVA tumour cells 14 days prior. Representative (left) and pooled data (right) are shown. *n* = 7 (WT) and 8 (cKO) mice per group from two independent experiments. *p*-value was calculated using a two-tailed unpaired *T* test, exact *p* value = <0.0001. Error bars depict SEM, *p < 0.05, **p < 0.01, ***p < 0.001.

To assess the role of *Egr2* in the CD8^+^ T cell exhaustion process, we utilised *Egr2* floxed mice crossed to a *Cd4*-cre transgene to delete the *Egr2* gene within all T cells^31^. *Cd4*-cre^+^ *Egr2*^f/f^ mice (cKO), or *Cd4*-cre^+^ *Egr2*^+/+^ littermates (WT), were infected with LCMV-Cl13 and the response to infection was assessed. There was a significant decrease in the number of GP_33–41_ and GP_276–286_-specific CD8^+^ T cells in cKO mice at day 20 p.i. (Supplementary Fig. 2a,b). Moreover, EGR2 loss diminished the expression of multiple inhibitory receptors (PD-1, 2B4 and TIM3) within tetramer+ CD8^+^ T cells over the course of infection, while CD160 and LAG3 were either unchanged or even elevated within cKO cells (Fig. 3a). This led to a reduced overall load of inhibitory receptor expression, with a significant reduction in the proportion of cKO cells expressing five or more inhibitory receptors, and a corresponding increase in cells with 2–3 inhibitory receptors (Fig. 3b). However, this alteration in inhibitory receptor expression was not accompanied by an alteration in function. IL-2 and TNFα production within virus-specific CD8^+^ T cells was largely unaltered in cKO mice (Supplementary Fig. 2a, c, d). We additionally examined cytotoxicity within WT vs cKO mice at day 20 p.i. using an in vivo cytotoxicity assay, and while there was diminished target killing, this was largely due to the diminished number of GP_33–41_-specific cells at this time-point (Supplementary Fig. 2b), suggesting killing capacity is largely intact on a per cell basis (Supplementary Fig. 2e). Consistent with these observations, viral control was unchanged in this model (Supplementary Fig. 2f). Thus, despite altered inhibitory receptor expression, effector functions were largely unchanged in the absence of EGR2.

### EGR2 controls key genes and promotes exhausted T cell differentiation

To globally map the EGR2-regulated molecular pathways that could influence inhibitory receptor expression, we performed RNA sequencing (RNAseq) analysis on GP33^+^ CD8^+^ T cells isolated from LCMV-Cl13 infected WT or cKO mice at day 20 p.i. We identified 415 differentially expressed genes (Supplementary Data 2; FDR < 0.05), including diminished expression of key genes associated with exhaustion (*Pdcd1*, *Tigit*, *Batf*, *Tox*, *Tox2*, *Cd200r1*, *Cd200r4*), coupled with an increase in progenitor exhausted cell genes (*Bcl6*, *Satb1*, *Bach2*, *Il7r*) (Fig. 3c). Notably, cKO cells had a marked enrichment of a published progenitor exhausted cell signature, and a corresponding loss of a signature associated with terminal exhaustion^11^ (Fig. 3d), although some key genes of progenitor exhausted cells, including *Cxcr5* and *Eomes*, opposed this trend, which we independently confirmed at the protein level (Fig. 3c, Supplementary Fig. 3a, b). Nevertheless, flow cytometric analysis validated that there was indeed a pronounced increase in the proportion of TCF1^+^TIM3^−^ progenitor cells from day 20 p.i. onwards in cKO mice (Fig. 3e). The relative increase in TCF1^+^TIM3^−^ progenitor cell proportion was due to a loss of TCF1^−^ exhausted cells rather than an expansion of progenitor cells. TCF1^+^ cell number was largely unchanged in cKO mice, whereas TCF1^−^ exhausted cell number was consistently decreased by 2–4 fold from day 20 p.i. onwards (Fig. 3f). In contrast to chronic LCMV infection, and consistent with the limited expression of EGR2 during acute LCMV infection, EGR2 loss had little impact upon effector and memory CD8^+^ T cell differentiation during LCMV-Arm infection. Cell numbers, effector and memory cell subsets, and marker expression (including markers altered in exhausted cKO cells such as PD-1) were largely unchanged within LCMV-specific CD8^+^ T cells across the course of infection in WT and cKO mice (Supplementary Fig. 4). Thus, EGR2 selectively controls exhausted CD8^+^ T cell differentiation.

Finally, we examined whether EGR2 plays similar roles in exhausted CD8^+^ TIL differentiation. To exclude the impact of *Egr2* loss in CD4^+^ T cells, we crossed *Egr2* floxed mice to a *Cd8*-cre transgene to delete *Egr2* selectively within peripheral CD8^+^ T cells (CD8 cKO)^32^ and inoculated the mice with B16-OVA cells. CD8 cKO mice exhibited no change in tumour growth, or PD-1^hi^CD8^+^ TIL density, relative to WT littermates (Supplementary Fig. 3c, d). However, analogous to LCMV, there was an increased proportion of progenitor exhausted TILs in CD8 cKO mice (Fig. 3g). Overall, these data demonstrate that EGR2 regulates multiple aspects of exhausted T cell differentiation, including expression of key molecules linked to TCF1^+^ progenitor exhausted cells and differentiation of TCF1^–^ exhausted T cells.

### EGR2 controls the exhausted CD8^+^ T cell response to checkpoint blockade

**Fig. 4:**
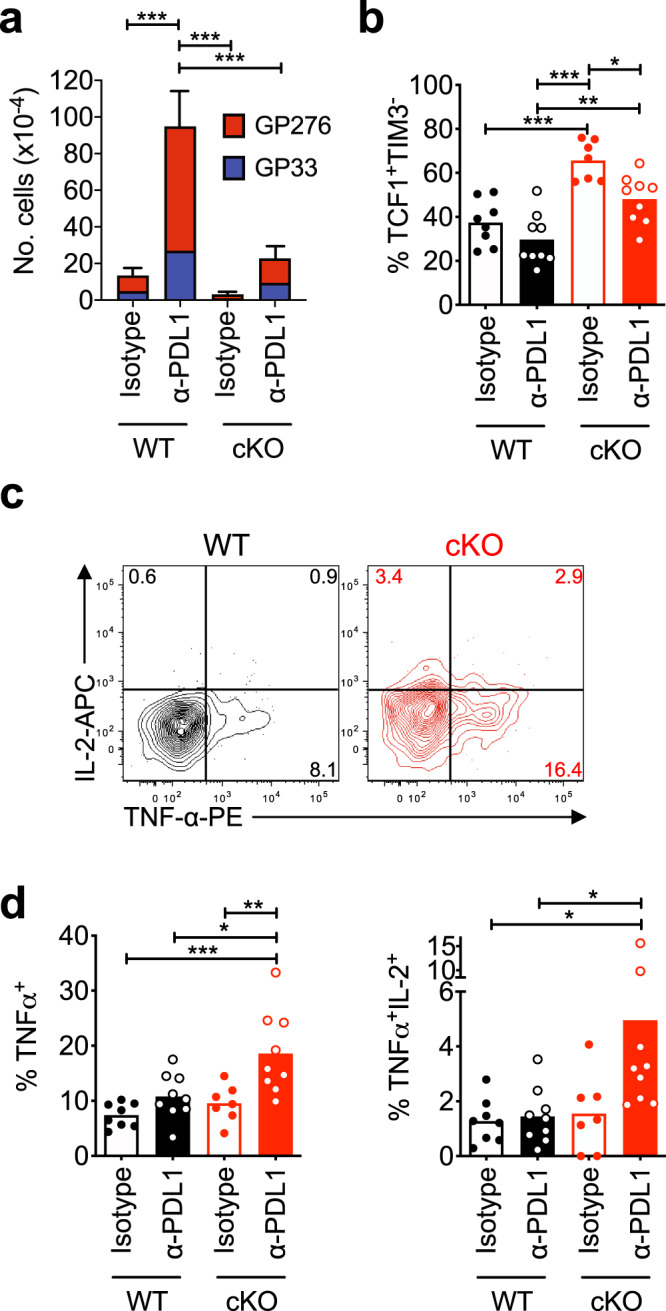
EGR2 regulates the exhausted CD8^+^ T cell response to checkpoint blockade. WT or cKO mice were depleted of CD4^+^ T cells then infected with LCMV-Cl13, and from days 28-42 p.i. mice were treated with anti-PD-L1 or isotype control antibody every 3 days prior to analysis at day 42 p.i. **a** The sum of GP_33–41_ and GP_276–286_ specific CD8^+^IFNγ^+^ T cell numbers (error bars show error of the summed numbers). For indicated significant differences, exact *p* values (left to right) = 0.0001, <0.0001, 0.0004. **b** %TIM3^-^TCF1^+^ cells within H2-D^b^GP_33–41_ tetramer-stained CD8^+^ T cells. For indicated significant differences, exact *p* values (left to right)= <0.0001, <0.0001, 0.0049, 0.0136. **c**, **d** Representative (**c**) and pooled (**d**) **%**TNFα^+^ and **%**TNFα^+^IL-2^+^ cells within the GP_276**–**286_ peptide-specific CD8^+^IFNγ^+^ T cells. *n* = 7–9 mice per group from three independent experiments. For indicated significant differences, exact *p* values (left to right) for left graph = 0.0003, 0.0102, 0.0048, for right graph = 0.0347, 0.0385. All *p*-values in this figure were calculated using a one-way ANOVA with a Tukey’s post-test. Error bars depict SEM, *p < 0.05, **p < 0.01, ***p < 0.001.

Checkpoint blockade triggers the proliferation and differentiation of TCF1^+^ progenitor cells, and this cell subset is a key mediator of the response to therapy^9,11,13,15,17,18^. We thus tested whether PD-L1 blockade could trigger expansion and differentiation of cKO exhausted cells. Despite having comparable numbers of TCF1^+^ cells to WT mice (Fig. 3f), cKO mice had a markedly impaired response to PD-L1 blockade. Virus-specific CD8^+^ T cell expansion was dramatically blunted, and therapy failed to increase the proportion of TCF1^−^ cells to the levels seen in WT mice after therapy (Fig. 4a, b). Fold expansion after therapy was similar in both contexts (~7.1 fold), however, given the comparable number of therapy-responsive TCF1^+^ cells in WT and KO mice prior to therapy, similar absolute numbers would be expected after therapy. Despite the blunted expansion, we did observe a marked rescue in cytokine polyfunctionality (i.e. IFNγ^+^TNFα^+^ and IFNγ^+^TNFα^+^IL-2^+^ cells) in GP276^+^ cKO vs WT cells after therapy (Fig. 4c, d), although the rescue in per cell IL-2 production (as evident by the low IL-2 MFI) was minimal. These data demonstrate that EGR2 is also required for therapy-induced expansion and differentiation of exhausted cells.

### EGR2 regulates exhausted cell maintenance and phenotype

**Fig. 5:**
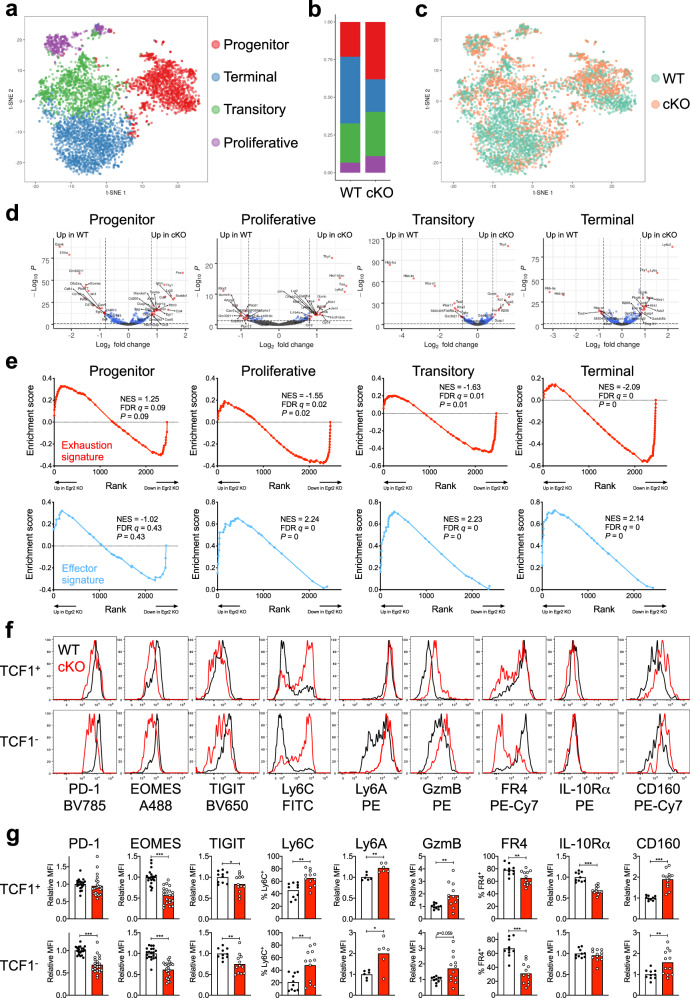
EGR2 loss leads to altered phenotypes in exhausted cell subsets. **a**–**e** H2-D^b^GP_33-41_ tetramer-stained CD8^+^ T cells were sorted from CD4-depleted, chronic LCMV infected WT or cKO mice at day 20 p.i., and subjected to multiplexed scRNAseq. **a** tSNE plot showing clusters identified within the pooled WT and cKO cells using published signatures. **b** Proportional distribution of the clusters identified in **a** within WT and cKO cells. **c** tSNE plot from **a** showing the location of WT (aqua) and cKO (orange) cells. **d** Volcano plots generated by comparing gene expression within cKO vs WT cells in each cluster from **a**. Dotted lines indicate *p*-value (0.05) and log_2_ fold change (>0.8 or <−0.8) cut-off values. Red dots indicate genes that satisfy both *p*-value and fold change cut-offs, while blue dots indicate genes that satisfy the *p*-value but not the fold change cut-off. Genes up in cKO are on the right, while genes up in WT are on the left. **e** GSEA analysis of the comparisons from **d** looking at enrichment of genes selectively upregulated in exhaustion (“Exhaustion signature”; upper plots in red), or genes selectively up in effector cells at day 8 of acute vs chronic LCMV (“Effector signature”; lower plots in blue). **f**, **g** Differentially expressed markers identified by the scRNAseq analysis in **d** were confirmed in H2-D^b^GP_33–41_ tetramer-stained CD8^+^ T cells from CD4-depleted, chronic LCMV infected WT or cKO mice at day 20 p.i. Representative plots (**f**) and pooled data (**g**) are shown. For the markers in **g**, WT *n* = 20 (PD-1, EOMES), 10 (TIGIT, Ly6C, GzmB, FR4, IL-10Rα, CD160), 6 (Ly6A), and KO *n* = 22 (PD-1, EOMES), 12 (TIGIT, Ly6C, GzmB, FR4, IL-10Rα, CD160), 6 (Ly6A), mice per group from 2–5 independent experiments. *p*-values were calculated using a two-tailed Mann–Whitney test (TCF1^-^ Ly6C, TCF1^-^ Ly6A, TCF1^+^ GzmB, TCF1^-^ GzmB, TCF1^+^ CD160) or a two-tailed unpaired *T* test (all others). For indicated significant differences, exact *p* values (left to right graphs) for TCF1^+^= <0.0001, 0.0466, 0^.^0014, 0.0015, 0.0056, 0.0070, <0.0001, <0.0001, for TCF1^−^= <0.0001, <0.0001, 0.0059, 0.0090, 0.0411, <0.0001, 0.0084. *p < 0.05, **p < 0.01, ***p < 0.001.

To define how EGR2 regulates exhaustion at high resolution, we sorted splenic GP33^+^ CD8^+^ T cells from LCMV-Cl13 infected WT or cKO mice at day 20 p.i. and conducted single-cell RNAseq (scRNAseq). Similar to other recent studies^15,33^, unbiased clustering of all cells on a tSNE plot generated 4 clusters that were annotated based on published scRNAseq gene signatures into “progenitor”, “proliferative”, “transitory” and “terminal” exhausted cells (Fig. 5a, Supplementary Fig. 5a). Consistent with our flow cytometry data, there was an enrichment of the progenitor cluster in cKO mice, however we also observed a more marked depletion of the terminal cluster relative to the other non-progenitor clusters (Fig. 5b, c). We independently confirmed this by flow cytometry, where there was a pronounced loss in percentage and number (~5 fold) of cKO vs WT terminally differentiated CD101^+^ cells^10^, although cKO CD101^-^ transitory exhausted cells were still decreased by ~2 fold (Supplementary Fig. 5b, c). These data support our previous observations that EGR2 controls the differentiation of exhausted cells.

To quantify gene expression differences, we conducted differential expression analysis between cKO and WT cells within each cluster. Multiple genes were differentially expressed in cKO vs WT cells in each exhausted cluster even after applying a fold change filter (Fig. 5d, Supplementary Data 3; Progenitor = 40 genes, Proliferative = 37 genes, Transitory = 20 genes, Terminal = 25 genes), including up-regulation of genes encoding chemokines and cytokines (*Ifng*, *Ccl3*, *Ccl4*, *Ccl6*) and effector-associated transcription factors (*Fos*, *Jun*, *Junb*, *Egr1*), and loss of progenitor cell-associated genes (*Cxcr5*, *Eomes*, *Sell*, *Il10ra*). Furthermore, a core set of genes were identified as differentially expressed in all four clusters, including induction of effector-associated genes (*Fos*, *Jun*, *Junb*, *Ly6c2*, *Id2*, *Gzmb*) and depletion of signature exhaustion genes (*Pdcd1*, *Tigit*, *Tox2*, *Cxcr5*) (Supplementary Data 3). Strikingly, GSEA of the three TCF1^−^ clusters using previously published exhaustion and effector gene signatures^3^ showed that exhaustion-associated genes were depleted in cKO cells while the effector signature was enriched (Fig. 5e). Thus, the TCF1^-^ cells in cKO mice are transcriptionally more effector-like. We then confirmed a number of these differences within cKO TCF1^+^ and TCF1^−^ tetramer^+^ cells, including diminished expression of EOMES, TIGIT and FR4 expression, and elevated expression of Ly6C, Ly6A, GzmB and CD160 (Fig. 5f, g). Consistent with the scRNAseq data, there were also subset-specific changes; IL10Rα was selectively lost from TCF1^+^ cells while PD-1 was more markedly lost from TCF-1^−^ cells (Fig. 5f, g). Thus, EGR2 stabilises the phenotype of TCF1^+^ progenitor exhausted cells, and maintains the exhausted cell identity of their TCF1^−^ progeny.

### EGR2 directly controls exhaustion genes and indirectly stabilizes the exhausted epigenetic state

**Fig. 6:**
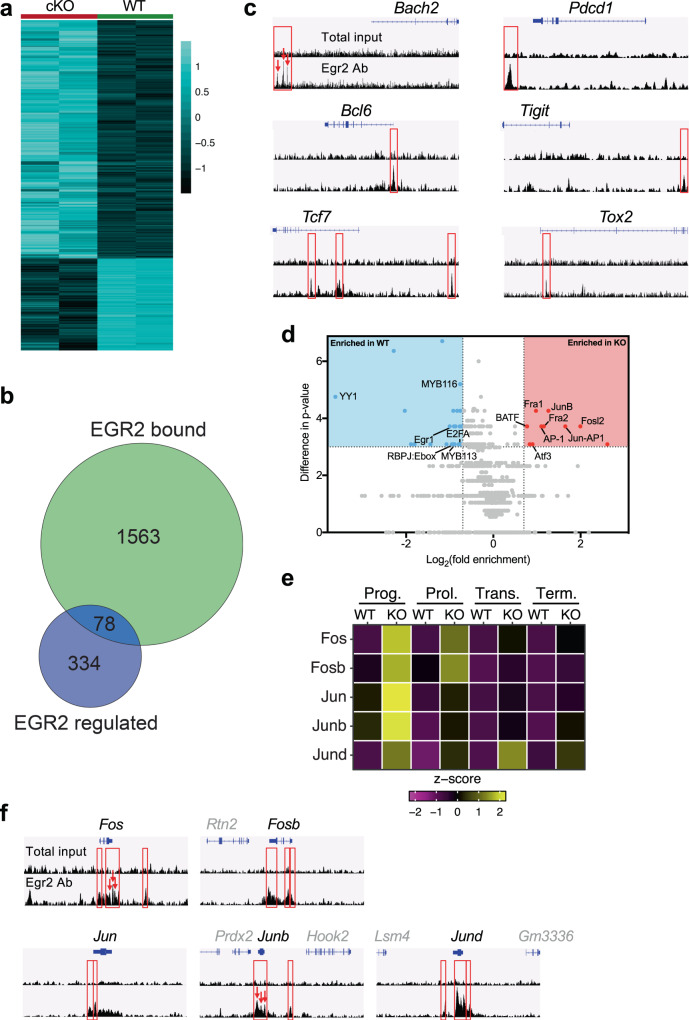
EGR2 directly regulates key exhaustion genes and indirectly stabilises the exhausted epigenetic state. **a** Z-score heatmap of differentially open regions (DORs; FDR < 0.01) identified by ATACseq between cKO (left) and WT (right) H2-D^b^GP_33–41_ tetramer-stained CD8^+^ T cells sorted from CD4-depleted, chronic LCMV infected WT or cKO mice at day 20 p.i. (*n* = 2 replicates per genotype). **b**, **c** CD8^+^ T cells were isolated from B6 mice at day 20 p.i. with LCMV-Cl13 and subjected to EGR2 ChIP-seq analysis. **b** Venn diagram summarizing the overlap between genes identified as EGR2 bound by ChIP-seq within CD8^+^ T cells isolated from LCMV-Cl13 infection (EGR2 bound) and genes differentially expressed within exhausted cKO vs WT cells from Fig. 3c (EGR2 regulated). **c** Representative profiles of EGR2 ChIP-seq peaks within genes repressed (left) or induced (right) by EGR2. Traces show EGR2 Ab signal and Total input signal. Red boxes (and arrows) denote significant peaks. **d** The top 50 WT or cKO-specific ATACseq peaks were analysed by HOMER to identify transcription factor motifs enriched above background, and the differential enrichment of these motifs was then measured between cKO vs WT peaks. Plot shows log_2_(fold enrichment) (*x*-axis), and difference in enrichment *p*-value (*y*-axis), between cKO vs WT. Dotted lines mark cut-offs for *p*-value difference (≥3) and log_2_(fold enrichment) (≥0.7 or ≤−0.7), while blue box (enriched in WT) and red box (enriched in KO) indicate enriched motifs that passed these cut-offs. **e** Z-score heatmap showing average AP-1 gene expression level within WT vs cKO cells in each exhausted cell cluster from the scRNAseq experiment in Fig. 5. **f** EGR2 binding to AP-1 genes. Red boxes (and arrows) denote significant peaks.

Exhausted cells adopt a distinct epigenetic state that plays important roles in the establishment and maintenance of exhaustion^2,4,22,34–39^. Given that EGR2 regulates TCF1^−^ cell number and phenotype despite TCF1^+^ cell-restricted expression, we speculated that EGR2 regulates T cell exhaustion through an epigenetic mechanism. To test this idea, we used ATACseq^40^ to map the open and closed chromatin regions within sorted virus-specific WT and cKO CD8^+^ T cells at day 20 p.i. with LCMV-Cl13. ATACseq analysis revealed 1876 differentially open regions (DORs) between WT and cKO cells (FDR < 0.01; Supplementary Data 4). Of these DORs, the majority were chromatin regions that were open in cKO cells (1353 regions with increased accessibility in cKO vs 523 regions with decreased accessibility in cKO)(Fig. 6a, Supplementary Data 4). Notably, of the 415 genes that we identified as differentially expressed in cKO vs WT cells (Fig. 3c), 67 genes were proximal to a DOR (Supplementary Table 1), including the cKO upregulated *Il12rb* gene and the cKO downregulated *Cd6* gene (Supplementary Fig. 6a, b). Importantly, most of these DORs were not accounted for by the increased proportion of TCF1^+^ cells in cKO mice, as few of the identified DORs overlapped with known TCF1^+^ vs TCF1^−^ cell-associated accessibility changes^41^ (Supplementary Fig. 6c, Supplementary Data 4). Thus, EGR2 controls the epigenetic state of exhausted CD8^+^ T cells.

To identify genes and chromatin regions directly controlled by EGR2, we next conducted ChIP-seq analysis of EGR2 binding within exhausted CD8^+^ T cells. CD8^+^ T cells were enriched from LCMV-Cl13 infected B6 mice at day 20 p.i., and the genome-wide pattern of EGR2 binding was assessed. A total of 2259 EGR2 binding peaks were identified (FDR < 0.05), with 1983 peaks (87.8%, associated with 1643 genes) found to localise to open chromatin regions. Unbiased de novo motif analysis identified highly significant enrichment of the EGR2 motif within these ChIP-seq peaks (Supplementary Table 2), with the identified motif closely matching the consensus EGR2 motif (Supplementary Fig. 6d). EGR2 binding sites were also highly significantly enriched in promoters and 5′UTRs (*p* < 0.0001 for both, Chi-square test with Yate’s correction)(Supplementary Fig. 6e). 78 genes were bound by EGR2 and differentially expressed within cKO cells (Fig. 6b, Supplementary Table 3; 50 repressed and 28 induced by EGR2), including key exhaustion-associated genes that are induced (*Pdcd1*, *Tigit*, *Tox2, Cxcr5*) and repressed (*Bach2*, *Bcl6, Tcf7*) by EGR2 (Fig. 6c, Supplementary Fig. 6f). Notably, the open chromatin region downstream of the *Pdcd1* gene bound by EGR2 has previously been shown to contribute to PD-1 expression^4^. Furthermore, a large number of the core genes either repressed or induced by EGR2 in all scRNAseq clusters (Supplementary Data 3) were direct EGR2 targets (specifically the EGR2 repressed genes *Fos*, *Jun*, *Junb*, *Thy1*, *Dusp1*, *Ly6c2*, and the EGR2-induced genes *Pdcd1*, *Tigit*, *Nab1*, *Tox2*, *Cxcr5*). Thus, EGR2 directly controls key exhaustion genes. Notably, only 4 of a high confidence set of 50 genes directly induced by EGR2 during in vitro CD4^+^ T cell anergy^27^ were shared direct targets during exhaustion (*Egr2*, *Bach2*, *Ryr1*, *Cd74*) (Supplementary Fig. 7a, Supplementary Table 3), suggesting classical anergy-associated EGR2 targets are not responsible for the observed phenotypes. Consistent with this observation, *Egr2* loss failed to rescue either the ERK or mTOR signalling pathways that are normally impaired by EGR2-induced target genes in anergic cells^26^ (Supplementary Fig. 7b, c).

We next examined whether EGR2 directly induced the chromatin accessibility changes observed in cKO cells. Strikingly, only 168 of the 1876 cKO associated DORs overlapped with an EGR2 binding site (Supplementary Fig. 6g, Supplementary Data 4), although the majority (124) of these EGR2-bound DORs were increased in accessibility within WT cells suggesting EGR2 may normally open these regions. This implies that most of the observed epigenetic changes must be through indirect regulation of other transcription factors by EGR2. TOX was recently identified as an important regulator of the exhausted epigenetic state^34,36–38^. Given that *Tox* expression is significantly decreased in cKO cells (Fig. 3c), we speculated that the observed changes were TOX-dependent. However, there was only minimal overlap between previously identified TOX regulated regions^36^ and DORs in cKO cells (185/1876 DORs; Supplementary Fig. 6h, Supplementary Data 4), suggesting that these effects are largely TOX-independent.

To identify transcription factors responsible for the observed changes in an unbiased manner, we examined transcription factor motif enrichment within WT and cKO-specific open chromatin regions. Strikingly, 8 of 10 of the most selectively enriched motifs within cKO-specific peaks were for AP-1 family transcription factors (Fig. 6d, Supplementary Table 4). This observation aligned with the increased expression of the AP-1 factors *Fos*, *Fosb*, *Jun*, *Junb* and *Jund* across multiple exhausted cell clusters (Fig. 6e, Supplementary Data 5). Overall AP-1 expression was highest in cKO progenitor exhausted cells (Fig. 6e), consistent with selective *Egr2* expression within this subset. Notably, these effects appeared due to direct repression of all 5 AP-1 genes by EGR2, as between 2-5 EGR2 ChIP-seq binding peaks per gene were identified within, or adjacent to, each of these 5 genes (Fig. 6f). Coupled with recent data showing that *Fosl2* over-expression diminishes terminal exhaustion^42^, these data suggest that direct EGR2-dependent global repression of AP-1 family transcription factors is the primary pathway responsible for EGR2-mediated epigenetic regulation.

## Discussion

In this study, we investigated whether the anergy-associated transcription factor EGR2 regulates CD8^+^ T cell exhaustion in both chronic viral infection and tumours. We demonstrate that EGR2 expression is selectively sustained in progenitor exhausted CD8^+^ T cells in an antigen-dependent manner, and that EGR2 is downregulated in more differentiated exhausted cell subsets. EGR2 both enables exhausted cell differentiation, and stabilizes the exhausted phenotype of all exhausted cell subsets, through both direct regulation of key exhaustion genes, and indirect stabilization of the exhausted epigenetic state. Thus, EGR2 is a transcriptional and epigenetic regulator of the exhausted state that plays key role in the maintenance of the response.

We show that EGR2 expression is maintained by chronic antigen encounter, and is highest within progenitor exhausted cells, before being lost as cells differentiate. It is unclear why EGR2 expression is lost as cells differentiate given that TCF1^−^ cells will still encounter antigen, which is the trigger for *Egr2* expression during LCMV. It may either be related to diminishing TCR signal strength as terminally exhausted cells upregulate inhibitory receptors, or increased access of TCF1^−^ cells to inflammatory signals that are known to inhibit *Egr2* expression^43^ within the red pulp of the spleen where there are higher viral loads^11^. Overall, these findings are significant as they identify EGR2 as a functionally relevant marker of progenitor exhausted cells within both tumours and chronic viral infection. Given the clinical relevance of the progenitor subset, EGR2 thus represents an important additional marker for the identification of progenitor cells.

EGR2 is required to generate TCF1^−^ cells, particularly the terminally exhausted subset of TCF1^−^ cells. This is surprising given that EGR2 is not expressed within TCF1^−^ cells, however this defect is likely downstream of phenotypic changes in *Egr2* deficient progenitor exhausted cells. In particular, cKO progenitor exhausted cells exhibited loss of key markers (*Cxcr5*, *Eomes*, *Il10ra*, *Tigit, Izumo1r*) and induction of other differentiation factors (AP-1 transcription factors, *Ly6c*, *Gzmb*, *Ly6a*, *Cd160*), illustrating that EGR2 directly influences progenitor cell phenotype. Most of these changes were carried through to their TCF1^-^ progeny, with TCF1^−^ cells additionally failing to upregulate PD-1 expression. Our ChIP-seq data indicate that some of these effects are direct, with EGR2 directly binding to key genes dysregulated in cKO cells, such as *Pdcd1*, *Tigit*, *Tox2*, *Cxcr5*, *Tigit*, *Bcl6*, *Bach2* and *Tcf7*. Direct effects of EGR2 on these loci may have lasting consequences for gene expression upon differentiation. However, EGR2 additionally stabilizes the global epigenetic landscape of exhausted cells, and this effect appears indirect and likely due to global repression of AP-1 transcription factor expression. Collectively, we thus propose that EGR2 stabilises the progenitor cell phenotype through induction of factors that either limit TCR signalling (e.g. *Tigit*) or transcriptionally regulate the exhausted state (e.g. *Eomes*, *Tox2*), combined with repression of antigen-induced AP-1 expression that would otherwise remodel the cell’s epigenetic state. Removing these regulatory pathways through EGR2 deletion generates progenitor cells only capable of producing progeny with a more “effector-like” phenotype, and these effector-like TCF1^−^ cells have a compromised capacity to persist. Consistent with this idea, the deficiency in TCF1^−^ exhausted cells was not evident until day 20 p.i., demonstrating that TCF1^−^ cells can be generated in these mice but have trouble persisting. This concept has been observed in TOX deficient cells, where aberrant differentiation towards an effector-like phenotype compromises long-term persistence^33,34,36–38^. Furthermore, effector-like cells generated early during chronic LCMV infection do not persist to later time-points^8^. EGR2 thus plays an important role in stabilizing the progenitor cell phenotype in a manner that has consequences for subsequent differentiation. Strikingly, EGR2 also programs the capacity of exhausted cells to expand upon checkpoint blockade, while simultaneously limiting therapy-induced cytokine production. This again fits with the idea that EGR2 loss leads to a more effector-like phenotype within exhausted cells at the expense of progenitor cell differentiation capacity.

Our findings also add to the emerging data on AP-1 transcription factor function during exhaustion. Collectively, our data indicate that EGR2 plays a non-redundant role in insulating exhausted cells against antigen-induced AP-1 expression, which in turn appears to play an important role in stabilizing the exhausted state. Other studies have yielded a range of results when examining AP-1 function during exhaustion. c-JUN over-expression within CAR T cells enhances anti-tumour function and persistence^44^, while increased c-JUN levels in *Nrp1*^−/−^ cells is associated with an increase in the progenitor exhausted cell population^45^. In contrast, *Fosl2* limits terminal exhaustion and, in certain contexts, compromises exhausted cell persistence^42^. More recently, the AP-1 antagonist BACH2^46^ was found to be essential for progenitor exhausted cell maintenance, with aberrant progenitor cell differentiation and attrition seen in the absence of BACH2^47,48^. Our data align more with the *Fosl2* and, to a lesser extent, *Nrp1*^−/−^ studies; in the context of *Egr2* deficiency, elevated AP-1 transcription factor expression is associated with diminished exhaustion and a proportional increase in progenitor cells due to compromised persistence of terminally exhausted cells. The differences between these studies may be due to the composition of the AP-1 transcription factors in each context. In the first study, global induction of AP-1 factors by a CAR construct was also associated with poorer outcomes^44^; it was only when c-JUN was selectively over-expressed that better persistence was achieved, suggesting that the ratio of different AP-1 components may alter the outcome. Similarly, the effects of BACH2 on progenitor cell maintenance appear predominantly due to effects on the AP-1 factor BATF^47,48^, which was not elevated in *Egr2*^−/−^ cells. Alternatively, the overall load of AP-1 transcription factors, and/or the point during exhaustion when they are expressed, may also be important contributors to the outcome. Given that EGR2 is not expressed within TCF1^−^ cells, further work is needed to determine whether other transcription factors play more prominent roles in AP-1 repression within these more differentiated cells.

Finally, we observed little overlap between the EGR2-regulated gene program in CD8^+^ T cell exhaustion vs CD4^+^ T cell anergy. However, there are significant caveats in this comparison. Given that we were comparing our results to EGR2 targets in in vitro CD4^+^ T cell anergy, the lack of overlap could be due to differences between CD4^+^ and CD8^+^ T cell anergy, in vitro vs in vivo differentiation models, or differences in TCR specificity/affinity. Further work is needed to directly compare EGR2 targets in in vivo CD8^+^ T cell anergy vs exhaustion to resolve these concerns. Moreover, these results are consistent with other studies examining EGR2 gene targets in different contexts within CD4^+^ T cells, where distinct gene targets are observed in different contexts. For example, EGR2 directly binds to and induces *Bcl6* expression in CD4^+^ T follicular helper (Tfh) cells and thereby contributes to Tfh differentiation^49^, but *Bcl6* is not an EGR2 target during CD4^+^ T cell anergy. In fact, given that we also observed EGR2 binding to the *Bcl6* locus during exhaustion, coupled with the known transcriptional parallels between Tfh and TCF1^+^ progenitor exhausted CD8^+^ T cells^9,11,14^, similar mechanisms may dictate EGR2 gene targets in both Tfh and CD8^+^ T cell exhaustion. More broadly, though, defining the genes targeted by transcription factors expressed in both anergy and exhaustion remains an important unresolved issue. As multiple other shared transcription factors functionally contribute to both exhaustion and anergy (e.g. NFAT and NR4A factors^22,24^), future studies examining the overlap in their respective gene targets in exhausted vs anergic CD8^+^ T cells are needed to better define the regulatory pathways engaged in these disparate states of negative regulation.

## Methods

### Mouse strains, adoptive transfer and infections

C57BL/6 (B6) and B6.SJL-*PtprcaPep3b*/BoyJ (CD45.1) mice were purchased from the Australian Phenomics Facility, ANU, Australia or the Walter and Eliza Hall Institute Kew Animal Facility, VIC, Australia. P14 transgenic^50^, Egr2-GFP^28^, *Cd8*-cre (also called E8I-cre) mice^32^ and C57BL/6 backcrossed *Cd4*-cre^+^ *Egr2* floxed^31^ mice have all been described previously. Mice were infected with 2 × 10^5^ pfu LCMV-Arm virus by intraperitoneal (i.p.) injection, or with 2 × 10^6^ pfu LCMV-Cl13 or LCMV-Cl13-V35A^29^ virus by intravenous (i.v.) injection. LCMV viral plaque assays were conducted as described previously^51^. For CD4 depletion, mice were injected with 200 µg GK1.5 antibody (BioXCell) on days −1 and +1 of the infection. For P14 transfer experiments, 2 × 10^3^ CD45.1^+^ P14 cells were transferred i.v. into CD45.2^+^ B6 mice one day prior to infection. For PD-L1 blockade, 200 µg anti-PD-L1 antibody (clone 10F.9G2, BioXCell) or Rat IgG2b isotype control (anti-KLH, clone LTF-2, BioXCell) were administered i.p. every 3 days from days 28 to 42 p.i. All animal work was in accordance with protocols approved by the ANU and Peter MacCallum Cancer Centre Animal Experimentation Ethics Committees, and current guidelines from the Australian Code of Practice for the Care and Use of Animals for Scientific Purposes.

### Flow cytometric analysis and cell sorting

Single-cell suspensions were prepared from spleens for flow cytometry by passing the cells through a 70 µm cell strainer, and then red blood cell lysing in 0.83% NH_4_Cl. Cell suspensions were stained in PBS containing 2.5% Foetal Calf Serum and 0.1% Azide on ice for 30 min. For CXCR5 staining, cells were stained with CXCR5 antibody for 45 min. at room temperature prior to staining on ice for other markers. To eliminate dead cells from the analysis, cells were also stained with Fixable Viability Stain 620 (BD Biosciences) or LIVE/DEAD Fixable Aqua Dead Cell Stain Kit (Life Technologies) according to manufacturer’s instructions. For intracellular staining, cells were fixed, permeabilised and stained using the Foxp3 Transcription Factor Staining Buffer Set (eBioscience) according to the manufacturer’s instructions. Samples were collected on a BD LSRII, Fortessa, X20 or Symphony flow cytometer (BD Biosciences) using FACSDiva software (BD Pharmingen, v8.0.1), with data analyzed using FlowJo Software (Tree Star).

### Phosphoflow and cytokine staining experiments

For phosphoflow analysis, splenocytes were restimulated with plate-bound anti-CD3 (clone 500A2, BD Biosciences; coated using 1 µg/ml antibody in PBS) in a flat-bottomed 96 well plate for 30 min. Cells were then fixed and permeabilised using the BD Phosflow Fix Buffer I and Perm Buffer III according to the manufacturer’s instructions. Cells were stained with the appropriate surface and phosphoflow antibodies for 1 h at room temperature in PBS containing 2.5% Foetal Calf Serum and 0.1% Azide. For cytokine staining, cells were restimulated with 0.1 µg/ml of the appropriate synthesised viral peptide (Biomolecular Resource Facility, ANU) in the presence of 3 µg/ml Brefeldin A (eBioscience) and CD107a antibody for 6 h, prior to surface staining then fixation with Biolegend Fixation Buffer, and intracellular cytokine staining in eBioscience Permeabilisation Buffer according to manufacturer’s instructions.

### Antibodies and tetramers used for flow cytometric analysis

The following antibodies were used for staining (purchased from Biolegend unless otherwise stated): CD8α (clone 53-6.7; PerCP (1/100, Cat. no. 100732), BV421 (1/200, Cat. no. 100738), BUV395 (1/200, BD Biosciences Cat. no. 563786) and BUV805 (1/200, BD Biosciences Cat. no. 563786)), CD44 (clone IM7; Pacific Blue (1/400, Cat. no. 103020), BUV737 (1/1000, BD Biosciences Cat. no. 612799)), CD107a (clone 1D4B; FITC (1/400, Cat. no. 121606)), IFNγ (clone XMG1.2; PE-Cy7 (1/2000, eBioscience Cat. no. 25-7311-41), TNFα (clone MP6-XT22; PE (1/2000, Cat. no. 506306)), IL-2 (clone JES6-5H4; APC (1/100, eBioscience Cat. no. 17-7021-82)), CD45.1 (clone A20; FITC (1/200, BD Biosciences Cat. no. 553775)), CD45.2 (clone 104; BUV395 (1/200, BD Biosciences Cat. no. 564616)), Ly6C (clone AL21; FITC (1/200, BD Biosciences Cat. no. 553104)), PD-1 (clone 29F.1A12; BV785 (1/100, Cat. no. 135225)), Tim3 (clone RMT3-23; BV605 (1/100, Cat. no. 119721)), 2B4 (clone 2B4; FITC (1/50, BD Biosciences Cat. no. 553305)), Lag3 (clone C9B7W; PE (1/100, Cat. no. 125208)), CD160 (clone 7H1; PE-Cy7 (1/100, Cat. no. 143010)), Egr2 (clone erongr2; PE (1/50, eBioscience Cat. no. 143010)), TCF1 (clone C63D9; unconjugated (1/100, Cell Signaling Technologies Cat. no. 2203S) followed by a secondary antibody (Goat anti-Rabbit Alexa594 (1/1000, Invitrogen Cat. no. A11012))), CXCR5 (clone 2G8; BV421 (1/25, BD Biosciences Cat. no. 562856)), Slamf6 (clone 13G3; PE (1/200, BD Biosciences Cat. no. 561540), BV421 (1/200, BD Biosciences Cat. no. 740090)), CD101 (clone Moushi101; PE-Cy7 (1/200, eBioscience Cat. no. 25-1011-82)), CD127 (clone A7R34; PE-Cy7 (1/100, Cat. no. 135014)), TIGIT (clone 1G9; BV650 (1/100, BD Biosciences Cat. no. 744213)), Ly6A/E (clone E13-161.7; PE (1/200, BD Biosciences Cat. no. 553336)), GzmB (clone GB11; PE (1/200, eBioscience Cat. no. GRB04)), FR4 (clone eBio12A5; PE-Cy7 (1/100, eBioscience Cat. no. 25-5445-82)), IL-10Rα (clone 1B1.3a; PE (1/100, Cat. no. 112706)), KLRG1 (clone 2F1; FITC (1/200, eBioscience Cat. no. 11-5893-82)), CD62L (clone MEL-14; APC-Cy7 (1/200, Cat. no. 104428)), Eomes (clone Dan11mag; Alexa488 (1/50, eBioscience Cat. no. 53-4875-82)), ppErk1/2 (clone 197G2; Alexa647 (1/100, Cell Signaling Technologies Cat. no. 13148S)), and pS6 (clone D57.2.2E; Alexa488 (1/100, Cell Signaling Technologies Cat. no. 4803S)). MHCI LCMV tetramers (1/50) were purchased from the Biomolecular Resource Facility, JCSMR, ANU.

### Tumour inoculation and TIL isolation

B16-OVA cells were a gift from J. Oliaro, and the parental B16-F10 line was originally obtained from ATCC. Cells were routinely tested to ensure that they were negative for mycoplasma contamination. Cells were not genetically authenticated, although we did validate black melanocyte colouring and OVA expression by both GFP expression and susceptibility to OT-I killing. For tumour inoculation, sex-matched recipient mice were shaved and 2 × 10^5^ OVA transgenic B16-F10 male mouse melanoma cells (B16-OVA) were injected sub-cutaneously on the mouse flank. For TIL analysis, isolated tumours were digested on a gentleMACS Dissociator (Miltenyi Biotec) at day 14 post-inoculation in C Tubes (Miltenyi Biotec) using the mouse Tumour Dissociation Kit (Miltenyi Biotec) and the program “37C_m_TDK_1” according to manufacturer’s instructions. Cells were then washed, resuspended in a 44% Percoll solution, underlaid with a 56% Percoll solution, then centrifuged on low brake. TILs were isolated from the interphase, red blood cell lysed in 0.83% NH_4_Cl, and then stained for flow cytometric analysis.

### In vivo cytotoxicity experiments

For the in vivo cytotoxicity assay^52^, pooled lymph nodes and spleens harvested from CD45.1 mice were red blood cell lysed in 0.83% NH_4_Cl. Cells were split in half, with half the cells pulsed in 0.3 µg/ml LCMV GP_33–41_ peptide at 37 ^o^C for 1 h, and the other half mock treated. Peptide pulsed cells were then labelled with a high CTV concentration (5 µM) while unpulsed cells were labelled with a low CTV concentration (0.5 µM) in media for 5 min at room temperature. Cells were then mixed together, and 2 × 10^7^ cells were injected per mouse. The ratio of CD45.1^+^ CTV^hi^ and CTV^lo^ cells was assessed within the spleen by flow cytometry 4 h after target injection. % lysis was calculated as follows: [1 – (r unprimed/r primed)] × 100, where “r”= %CTV^hi^ / %CTV^lo^.

### RNAseq analysis

For Egr2-GFP RNAseq experiments (Supplementary Fig. 1), Egr2-GFP mice were infected with LCMV-Cl13 (8 mice per sort), and at day 20 p.i. GFP^+^ (top 50% of cells) and GFP^-^ (bottom 30% of cells) polyclonal CD8^+^CD44^int-hi^PD-1^+^Slamf6^+^Tim3^-^ cells were sorted from pooled splenocytes. ~2 × 10^5^–3 × 10^5^ cells were recovered per sort, with 2 separate sorts conducted (i.e. 4 samples in total). For Egr2 cKO RNAseq experiments (Fig. 3), CD4-depleted WT or cKO mice were infected with LCMV-Cl13 (~5 mice per genotype per sort). At day 20 p.i., CD8^+^CD44^int-hi^ H-2D^b^GP_33–41_-tetramer-stained cells were isolated by fluorescence-activated cell sorting, with two separate sorts conducted per genotype (i.e. four samples in total), and ~1 × 10^5^–2 × 10^5^ cells recovered per sort. Total RNA was isolated using Trizol LS (Thermo Fisher Scientific) according to manufacturer’s instructions, and RNAseq libraries were prepared at the Molecular Genomics Core Facility, Peter MacCallum Cancer Centre, using the Illumina TruSeq Stranded mRNA Library Prep Kit. Samples were sequenced on an Illumina NextSeq500 instrument with 75 bp single-end reads. All reads were aligned to the mouse genome reference (GRCm38/mm10) sequence using HiSat2^53^ with default parameters. Read counts were then generated for each gene in each sample using FeatureCounts from the Subread package (v1.5.0-p3)^54^ by using annotated gene locations. Differential expression analysis was performed using LIMMA (v3.32.4). An adjusted p-value threshold of 0.05 was used to identify significantly differentially regulated genes, with ggplot2 (v2.2.1) used for generation of volcano plots. Gene Set Enrichment Analysis^55^ was conducted by searching the fold change ranked cKO versus WT RNAseq or scRNAseq, or GFP^+^ versus GFP^-^ RNAseq, dataset against the genes from a published LCMV exhaustion signature^3^, a published signature of progenitor (CXCR5^+^) vs terminally exhausted (CXCR5^−^) T cells during chronic LCMV^11^, or published CD69^+^ and CD69^-^ progenitor signatures^7^. For the Man et al. dataset, the “Exhaustion signature” in Fig. 5 was derived from the gene list in Table S1 of the paper, which was filtered to only include genes with a FC of either >2 or <0.5 in the following four comparisons of WT RNAseq datasets: d8 Chronic vs d8 Acute, d8 Chronic vs Naïve, d30 Chronic vs d30 Acute and d30 Chronic vs naive. From the resulting gene list, genes up in the d30 Chronic vs d30 Acute comparison were used for the Exhaustion signature. For the “Effector signature” in Fig. 5, genes with a FC of <0.4 were used in the d8 Chronic vs d8 Acute comparison. For the signatures in Fig. 3 (Im et al. dataset), genes with a logFC of >2 (CXCR5^-^ terminally exhausted signature) or <−2 (CXCR5^+^ progenitor exhausted signature) in the CXCR5^−^ vs CXCR5^+^ comparison were included. For the Beltra et al. dataset, genes up in the “Texprog1 vs Texprog2” or “Texprog2 vs Texprog1” comparisons in Table S1 were used to create the CD69^+^ and CD69^-^ progenitor signatures respectively.

### scRNAseq analysis

CD4-depleted WT or cKO mice were infected with LCMV-Cl13 (~3 mice per genotype), and at day 20 p.i., CD8^+^CD44^int-hi^ H-2D^b^GP_33-41_-tetramer-stained cells were isolated by fluorescence-activated cell sorting. Prior to scRNAseq with feature technology (10x Genomics), cells were carefully counted (viability was registered at >95%) and resuspended in 1× Cell Staining Buffer (2% BSA, 0.01% Tween in PBS^56^, at ~1000 cells/µl) and then incubated for 10 min at 4 °C with TruStain FcX™ PLUS Blocking reagent (Biolegend). WT and cKO cells were then labelled with the Biolegend TotalSeq™-C0301 anti-mouse Hashtag 1 and TotalSeq™-C0302 anti-mouse Hashtag 2 antibodies, respectively, at 1 µg/sample for 30 min at 4 °C (as per manufacturer’s recommendations). After staining, cells were washed three times in PBS containing 2% BSA and 0.01% Tween, and after the final wash, cells were resuspended in PBS + 0.04% BSA and filtered through 40 µm cell strainers. Viability was determined (>90%), and cells were counted and resuspended at a density of 1500 cells/µl. Cell capture and library preparations were performed using the 10x Genomics Chromium system, the Chromium Single Cell 5′ Library & Gel Bead Kit (v1.0 chemistry) and the Chromium Single Cell 5′ Feature Barcode Library Kit. Single-cell and feature (hashtag) libraries were generated using the Chromium Controller and Single Cell 5′ Library & Gel Bead Kit and Chip A Kit (10x Genomics) according to the manufacturer’s protocol. Briefly, ~15,000 cells were gently mixed in Reverse transcription pre-mix and loaded onto the microfluidic Chip A. Reverse transcription to generate barcoded cDNA library for each cell was performed in the resultant Gel-bead-in-EMulsions (GEMs). Subsequently, single-cell droplets were broken, and the single-strand cDNA and hashtags were cleaned up using Dynabeads MyOne SILANE magnetic beads. Endogenous cDNA and hashtags were then amplified using SC5ʹ Feature cDNA Primers (from the feature barcode Library Kit) using the recommended cycling protocol based on the targeted recovery. Amplified endogenous cDNA and hashtags were then separated by size-selection using SPRI beads. Endogenous cDNA was analysed and quantified using a Bioanalyzer (Agilent), and 50 ng of cDNA was used for library preparation following manufacturer’s recommendations. Hashtag libraries were prepared as recommended by the manufacturer, namely indexed using Chromium i7 Sample indices N (Set A) after 9 cycles of amplification. Libraries were QCed and quantitated using the Bioanalyzer and Qbit instruments prior to loading onto a NovaSeq6000 instrument for paired end, 150 bp read sequencing (1% PhiX). >50,000 read-pairs per cell for gene expression libraries and >5000 read-pairs per cell for feature (hashtag) libraries were recovered. Cell Ranger v3.1.0 (10x Genomics), with reference genome GRCm38/mm10, and Ensembl gene annotation release 93, was used to generate gene×cell and hashtag×cell UMI count matrices from the raw sequencing reads. Downstream bioinformatic analysis was performed in R using packages downloaded from Bioconductor release 3.10. Low quality cells were filtered out by removing cells with a low number of detected genes (≤750), a low total UMI count (<1123) or a high percentage of reads aligning to mitochondrial genes (≥10%). Likely doublets (10× beads containing multiple cells) were removed based on hashtag proportions (≥8% and ≤96% wild-type) or high number of detected genes (>3250). Gene expression was normalised using the deconvolution method^57^. Highly variable genes (HVGs) were detected by fitting a mean variance trend and selecting genes with significantly greater variance than the trend using a chi-squared test. The following genes were excluded from further analysis: non-HVGs, genes expressed in <1% of cells and T cell receptor genes. The resulting dataset contained 2787 wild-type cells, 1960 knock-out cells and 2162 genes. The cells were clustered using the multi-level modularity optimization algorithm for finding community structure^58^. Differential expression was performed using EdgeR (v3.28.0)^59,60^. For the heatmap (Supplementary Fig. 5a), each gene was assigned to the cluster in which it was most highly expressed, and top genes for each cluster were plotted in the heatmap sorted by the maximum fold-change to any other cluster.

### ATACseq analysis

CD4-depleted WT or cKO mice were infected with LCMV-Cl13 (~2–3 mice per genotype), and at day 20 p.i., CD8^+^CD44^int-hi^ H-2D^b^GP_33-41_-tetramer-stained cells were isolated by fluorescence-activated cell sorting (two replicates per genotype, four samples in total). ATACseq library preparation was conducted based on a published protocol^40^. Briefly, cells were washed once in ice cold PBS, then resuspended in 50 µl ice-cold lysis buffer per 5 × 10^4^ cells (10 mM Tris-HCl pH 7.4, 10 mM NaCl, 3 mM MgCl_2_, 0.1% NP-40). Chromatin was then tagmented in 50 µl tagmentation mix (2.5 µl TDE1 Enzyme, 25 µl TDE Buffer (Illumina), and 22.5 µl H_2_O). Samples were incubated at 37^o^C for 30 min. after which tagmented chromatin was purified using a MinElute kit (Qiagen) according to manufacturer’s instructions. Tagmented DNA was amplified using previously reported indexing primers^40^ in a 50 µl PCR reaction (20 µl eluted DNA, 2.5 µl each of 25 µM Forward and Reverse primers and 25 µl of 2× KAPA HiFi Hotstart ReadyMix (Roche)). PCR amplification was conducted as follows: 72 ^o^C for 5 min., 98 ^o^C for 3 min, then 13 cycles of 98 ^o^C for 30 s, 65 ^o^C for 15 s, 72 ^o^C for 60 s. Amplified DNA was purified using a MinElute kit (Qiagen), after which the DNA was run on a TapeStation (Agilent Technologies) for quality control of tagmentation and to quantitate DNA concentration. Libraries were sequenced by 75 bp paired-end sequencing to a depth of ~60–80 × 10^6^ reads per sample on a NextSeq500 (Illumina). Sequenced ATACseq reads were trimmed with Trim_Galore version 0.4.5_dev, and aligned to GRCm38.p6 using BWA-MEM (v0.7.17)^61^ in paired-end mode with default parameters. The resulting bam files were deduplicated with MarkDuplicates (Picard) v2.19.0. Prior to further analysis, sample quality was verified using the ATACseqQC package (v1.10.1)^62^. Differentially open regions (DORs) were called between the Egr2 cKO and WT groups using csaw (v1.20.0)^63^ with a FDR < 0.01. Overlap with differentially expressed genes was defined as a DOR occurring within 20 kb of a differentially expressed gene locus. Motif enrichment over background for DORs was performed for the top 50 cKO and WT peaks separately using HOMER. For the plot in Fig. 6d, the log of the fold motif enrichment between WT and cKO peaks was plotted against the absolute value of the difference in *p*-values (calculated by plotting p-values first as a z-score, then measuring the z-score difference between WT and cKO). The plot in Fig. 6d was prepared using Prism Software (Version 8.0, GraphPad software). ATACseq tracks were visualized using IGV (v2.3.55). TOX DORs (Supplementary Fig. 6h) were previously published^36^ and were obtained from Supplementary Table 5 of the paper. TCF1^+^ and TCF1^−^ cell-specific ATACseq peaks were obtained by reanalysis of sequencing data from a previously published dataset^41^ using the above pipeline. Peaks were filtered on FDR < 0.001 as in the paper, and a logFC > 2 between the cell types.

### ChIP-seq analysis

B6 mice were infected with LCMV-Cl13, and at day 20 p.i. spleen and lymph nodes were isolated from the mice and pooled. CD8^+^ T cells were then isolated by MACS enrichment (Miltenyi) according to manufacturer’s instructions to yield ~93% purity of CD8^+^ T cells. Briefly, cells were labelled with CD8-PE antibody then enriched with anti-PE beads. Bulk CD8^+^ T cells were used in this analysis as we had previously confirmed by flow cytometry that, within total CD8^+^ T cells during LCMV-Cl13 infection, EGR2 protein is only expressed within PD-1^+^ CD44^int-hi^ exhausted CD8^+^ T cells. After enrichment, ~60 × 10^6^ CD8^+^ T cells from ~8 mice were then used for subsequent ChIP-seq analysis. Cells were resuspended in PBS and crossed-linked with fresh formaldehyde solution (Supplementary Table 5; 1× final concentration) for 30 min at room temperature. Excess formaldehyde was quenched with 125 mM Glycine for 5 min before cells were washed once with ice-cold PBS. Cells were incubated three times with nuclear extraction buffer (Supplementary Table 5) containing Roche cOmplete protease inhibitors (MERCK, 04693159001) on ice for 5 min. Nuclear extracts were resuspended in sonication buffer (Supplementary Table 5) containing protease and Pierce phosphatase inhibitors (ThermoFisher Scientific, A32957) and sonicated using the Covaris S220 Focused Ultrasonicator for 18 min. Sheared lysates were diluted with 1 volume of ChIP dilution buffer (Supplementary Table 5) containing protease and phosphatase inhibitors. Protein A and Protein G Dynabeads (ThermoFisher Scientific, 10002D and 10004D) were mixed 1:1 (50 µl total per IP) and washed in blocking buffer (Supplementary Table 5) containing protease inhibitors at 4 ^o^C. Protein A/G beads were resuspended in ChIP IP buffer (Supplementary Table 5; ~0.5 mL/IP) containing protease and phosphatase inhibitors and added to diluted lysates with 20 µg of a previously validated polyclonal anti-EGR2 ChIP antibody (ab43020, Abcam)^64^ and 0.45% bovine serum albumin (BSA). IP samples were incubated overnight at 4^o^C while tumbling. Protein A/G beads were washed twice with ChIP IP buffer on ice, before washing once with ChIP Wash Buffer 1 and Wash Buffer 2 (Supplementary Table 5), each containing protease and phosphatase inhibitors, and washing twice with Tris-EDTA buffer (10 mM Tris-HCl pH 7.5, 1 mM EDTA). Washed beads were incubated with Reverse Crosslinking Buffer (Supplementary Table 5) containing 45 μg Proteinase K at 55 ^o^C for 1 h and the supernatant was then isolated and incubated at 65^o^C overnight. DNA was isolated using the ChIP DNA Clean & Concentrator Kit (Zymo Research, D5205). Indexed libraries were prepared using KAPA Hyper Prep Kit for Illumina platforms (Kapa Biosystems) and the SeqCap Adapter Kit (Roche) following vendor’s instructions. Library QC and quantification was performed using D1000 high-sensitivity screen tape with a 4200 TapeStation Instrument (Agilent Technologies) and size selected for between 200 and 500 bp using a Pippin Prep system (Sage Science). Libraries were pooled and sequenced with 75 bp single-end sequencing to a depth of 15–20 × 10^6^ reads per sample on a NextSeq500 (Illumina). bcl2fastq (v2.17.1.14) was used for de-multiplexing. The Fastq files were then aligned to the mouse reference genome (GRCm38/mm10) using bowtie2 (v2.3.3). Samtools (v1.4.1) was used for the manipulation of SAM and BAM files. MACS (v2.1.1) was used for peak-calling, and significant peaks (FDR < 0.05) were then associated with the closest gene TSS using Bedtools (v2.26). Motif analysis was performed with HOMER (v4.8). Browser viewable TDF files were generated using IGVTools (v2.3.95) and ChIP-seq tracks were visualized using IGV (v2.3.55).

### Statistical analysis

Data calculations were conducted in Microsoft Excel (v14.7.2) while data were graphed and statistical analysis conducted using Prism (GraphPad, v8.1.2). *P* values were calculated using a two-tailed unpaired *T* test or a two-tailed Mann–Whitney test when data failed normality tests. Where >2 conditions were compared, a one-way ANOVA with a Tukey post-test was used to calculate *P* values. Where indicated in the text, a Chi-square test with Yate’s correction or Fisher’s Exact test was used to calculate enrichment *P* values.

### Reporting summary

Further information on research design is available in the Nature Research Reporting Summary linked to this article.

## Supplementary information

Supplementary Information

Description of Additional Supplementary Files

Supplementary Data 1

Supplementary Data 2

Supplementary Data 3

Supplementary Data 4

Supplementary Data 5

Reporting Summary

## Data Availability

The RNAseq, scRNAseq, ATACseq and ChIP-seq sequencing datasets generated during the current study associated with Figs. 3, 5 and 6, and Supplementary Fig. 1 are available in the NCBI GEO repository at accession number GSE134710. The gene lists used for GSEA analysis in Supplementary Fig. 1d were extracted from Table S1 in the cited Beltra et al. study (available at 10.1016/j.immuni.2020.04.014). The gene lists used for GSEA analysis in Fig. 3 were taken from the publicly available dataset GSE84105 within the NCBI GEO database. The gene lists used for GSEA in Fig. 5 were extracted from supplementary data in the cited Man et al study (available at 10.1016/j.immuni.2017.11.021). TOX DORs (Supplementary Fig. 6h) were obtained from Supplementary Table 5 of the cited Khan et al. paper. TCF1+ and TCF1− cell-specific ATACseq peaks were obtained by reanalysis of sequencing data from the cited Jadhav et al paper from the publicly available dataset PRJNA546023 within the NCBI BioProject database. All sequencing data were mapped and annotated using reference genome GRCm38/mm10 (https://www.ncbi.nlm.nih.gov/assembly/GCF_000001635.20/) and Ensembl gene annotation release 93 (http://ftp.ensembl.org/pub/release-93/).
