# Supplementary material for: Antigen-driven EGR2 expression is required for exhausted CD8+ T cell stability and maintenance
Source: Nat Commun. 2021 May 13;12:2782. doi: 10.1038/s41467-021-23044-9 (PMC8119420; doi:10.1038/s41467-021-23044-9)
Supplement: Supplementary file 1 — Supplementary Information [file 41467_2021_23044_MOESM1_ESM.pdf]

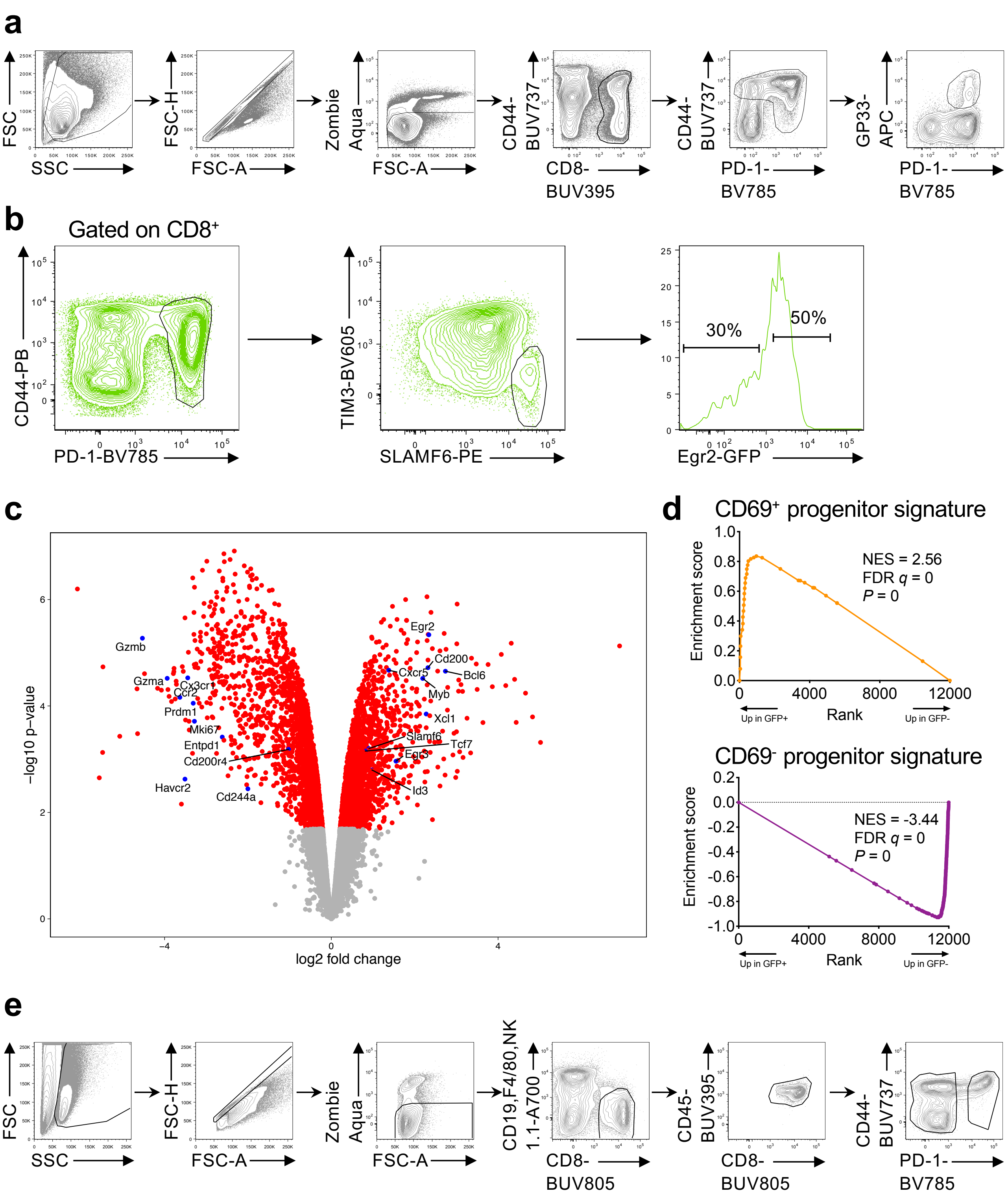

Supplementary Figure 1. EGR2 negative progenitor exhausted cells have a more differentiated phenotype. (a) Tetramer gating strategy used in LCMV throughout this study (Fig. 1a-c, 3a, 3e, 5f-g, and Supplementary Fig. 3a-b, 5b). A similar gating strategy was also used in Fig. 2 with GP33-APC tetramer replaced with Ly5.1-FITC antibody to identify P14 cells. **(b-d)** GFP$^+$ and GFP$^-$ polyclonal CD8$^+$CD44$^{int\text{-}hi}$PD-1$^+$Slamf6$^+$Tim3$^-$ cells were sorted at day 20 p.i. as in **(b)** and analysed by RNAseq (n=2 replicates per condition). Volcano plot showing differential gene expression in GFP$^+$ vs GFP$^-$ cells **(c)** and GSEA plots examining enrichment of published CD69$^+$ (orange) and CD69$^-$ (purple) progenitor cell signatures **(d)** are shown. Enrichment score p-values were calculated by the GSEA program using an empirical phenotype-based permutation test procedure. **(e)** TIL gating strategy used in Fig. 1d,e and 3g.

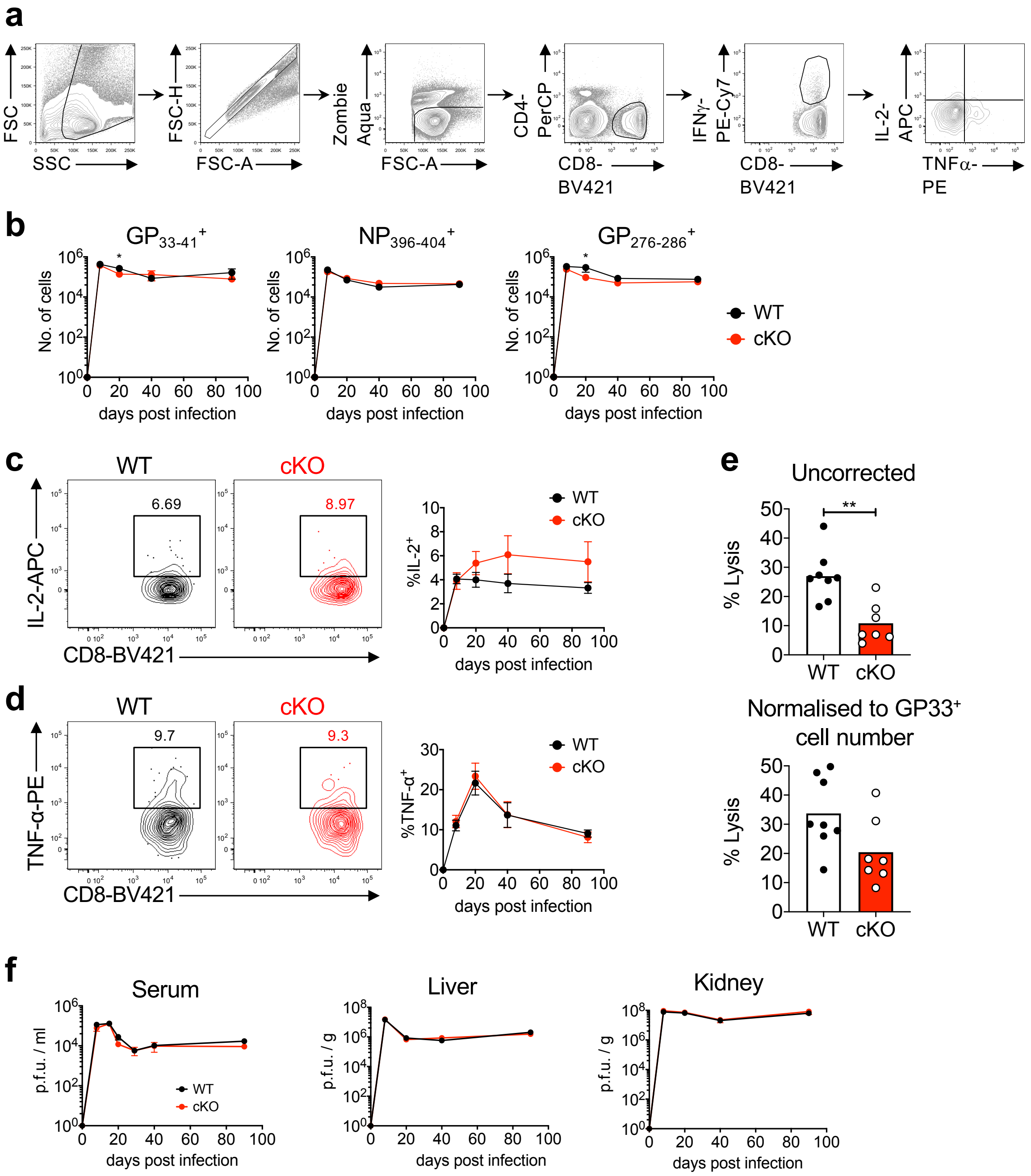

Supplementary Figure 2. CD8^{+} T cell expansion and function in LCMV-Cl13 infected WT vs cKO mice. (a-f) WT or cKO mice were depleted of CD4^{+} T cells then infected with LCMV-Cl13 and the response tracked over time. **(a)** Cytokine gating strategy used in this figure and Fig. 4c. CD8^{+} T cells were similarly gated in Supplementary Fig. 7b,c. **(b)** The number of CD8^{+}IFNγ^{+} T cells recovered over time upon stimulation of splenocytes with the indicated LCMV peptides. n=7-13 mice per group per time point from 2-3 independent experiments. p-values were calculated using a two-tailed Mann-Whitney test. For indicated significant differences, exact p values for GP_{33-41}^{+} = 0.0288, for $GP_{276-286}^{+}$ = 0.0115. **(c,d)** Representative (left; day 20 p.i.) and pooled (right) TNFα **(d)** and IL-2 **(c)** production within the GP_{33-41} peptide-specific CD8^{+}IFNγ^{+} T cells from (a) over time. **(e)** Specific lysis of GP_{33-41} coated targets within WT or cKO mice at day 20 p.i. Top graph shows absolute, uncorrected data per mouse, while bottom graph shows lysis normalized to the number of H2-D^{b}GP_{33-41} tetramer stained CD8^{+} T cells within each mouse. n=7-8 mice per group from 2 independent experiments. p-value was calculated using a two-tailed unpaired T test. For the indicated significant difference, exact p value = 0.0014. **(f)** Viral titres in serum, liver and kidney over time. n=5-13 mice per group per time point from 2-3 independent experiments. All error bars depict SEM. * = $p<0.05$, ** = $p<0.01$.

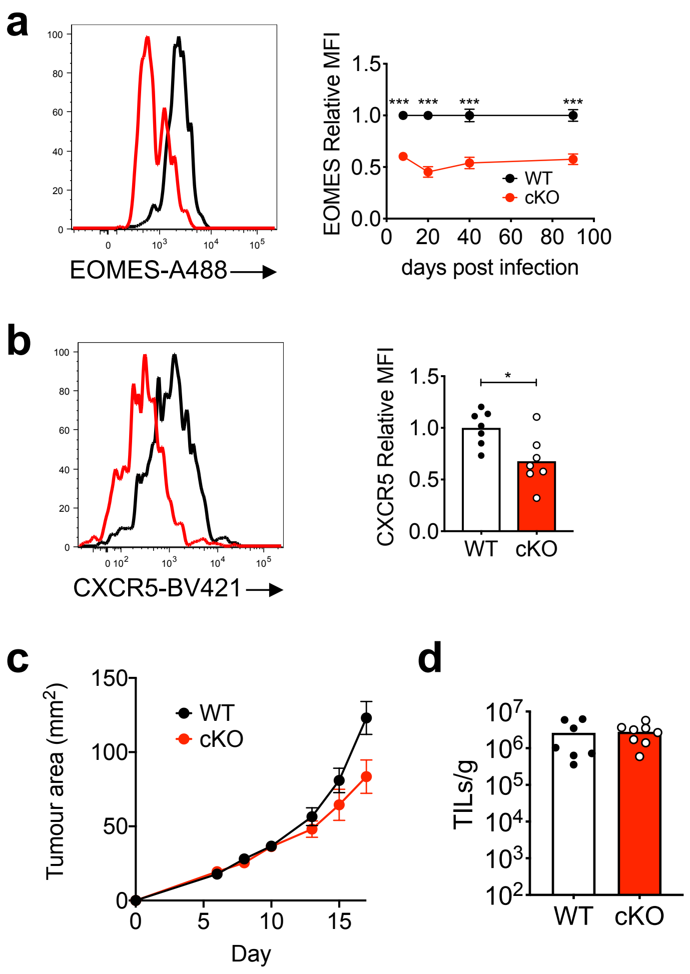

Supplementary Figure 3. Exhausted CD8$^+$ T cell and tumour phenotype in WT vs cKO mice. (a) EOMES levels over time within H2-D^{b}GP$_{33\text{-}41}$ tetramer+ cells in LCMV-Cl13 infected CD4-depleted WT or cKO mice (bottom; normalized to WT mean at each time-point) with representative day 20 p.i. histogram (top). n=7-13 mice per group per time point from 2 independent experiments. p-values were calculated using a two-tailed unpaired T test. For indicated significant differences, exact p values (left to right) = <0.0001, <0.0001, 0.0001, <0.0001. **(b)** CXCR5 MFI in H2-D^{b}GP$_{33\text{-}41}$ tetramer+ cells from LCMV-Cl13 infected CD4-depleted WT and cKO mice at day 40 p.i. n=7 mice per group from 2 independent experiments. p-value was calculated using a two-tailed unpaired T test, exact p value = 0.0140. **(c,d)** WT and CD8 cKO mice were given 2x10^5 B16-OVA cells s.c. and both tumour growth over time within each mouse **(c),** and PD-1^{hi} TIL density at day 14 post-inoculation **(d),** was measured. n=13 mice per group from 3 independent experiments for (b) and n=7-8 mice per group from 2 independent experiments for (c). All error bars depict SEM. * = p<0.05, *** = p<0.001.

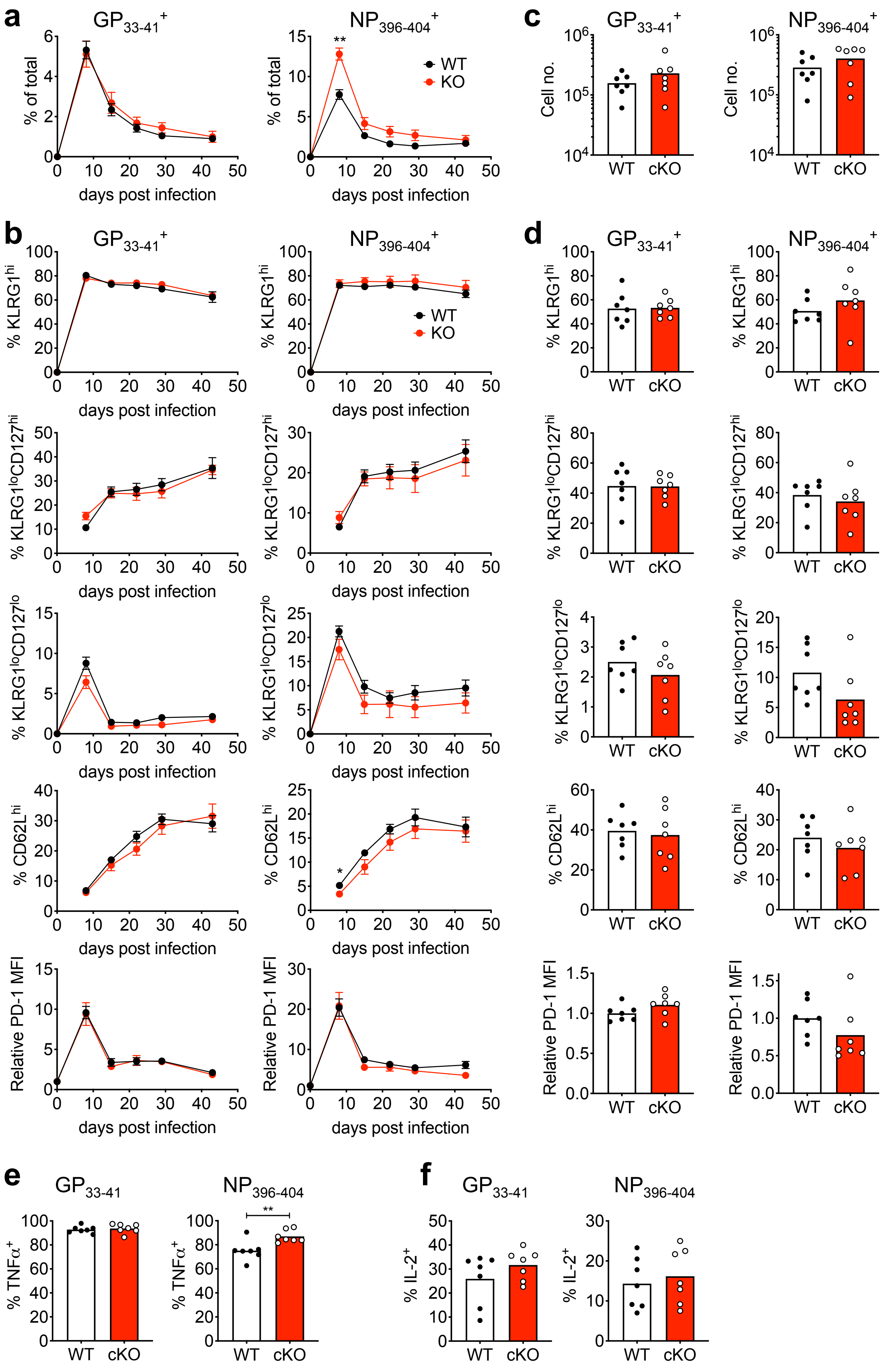

a
GP33-41+
NP396-404+
% of total
days post infection
WT
KO
**
c
Cell no.
WT
cKO
b
% KLRG1hi
% KLRG1loCD127hi
% KLRG1loCD127lo
% CD62Lhi
Relative PD-1 MFI
*
d
e
GP33-41
NP396-404
% TNFα+
f
% IL-2+

Supplementary Figure 4. CD8$^+$ T cell expansion and phenotype in LCMV-Arm infected WT vs cKO mice. WT or cKO mice were infected with LCMV-Arm and the response tracked over time within each mouse. **(a)** The proportion of total blood cells that were either H2-$D^b GP_{33\text{-}41}$ (left) or H2-$D^b NP_{396\text{-}404}$ (right) tetramer$^+$ CD8$^+$ T cells over time. p-values were calculated using a Mixed effects model with the Geiser-Greenhouse correction and a Sidak's multiple comparisons test. For the indicated significant difference, exact p value = 0.0027. **(b)** Marker expression within the cells in (a) over time. PD-1 MFI is expressed relative to naïve (CD44^{lo}) CD8$^+$ T cell MFI at each time-point. p-values were calculated as in (a). For the indicated significant difference, exact p value = 0.0320. **(c)** The number of splenic H2-$D^b GP_{33\text{-}41}$ (left) or H2-$D^b NP_{396\text{-}404}$ (right) tetramer$^+$ CD8$^+$ T cells at day 43 p.i. from the mice in (a,b). **(d)** Marker expression within the cells in (c). PD-1 MFI is expressed relative to naïve (CD44^{lo}) CD8$^+$ T cell MFI. **(e,f)** TNFα **(e)** and IL-2 **(f)** production within splenic $GP_{33\text{-}41}$ (left) or $NP_{396\text{-}404}$ (right) peptide-specific CD8$^+$IFNγ^+ T cells at day 43 p.i. n=6 (d8, both genotypes) or 7 (all other timepoints for both genotypes) mice per group per time point from 3 independent experiments. For the indicated significant difference, p-value was calculated using a two-tailed unpaired T test, exact p value = 0.0071. All error bars depict SEM. * = $p<0.05$, ** = $p<0.01$.

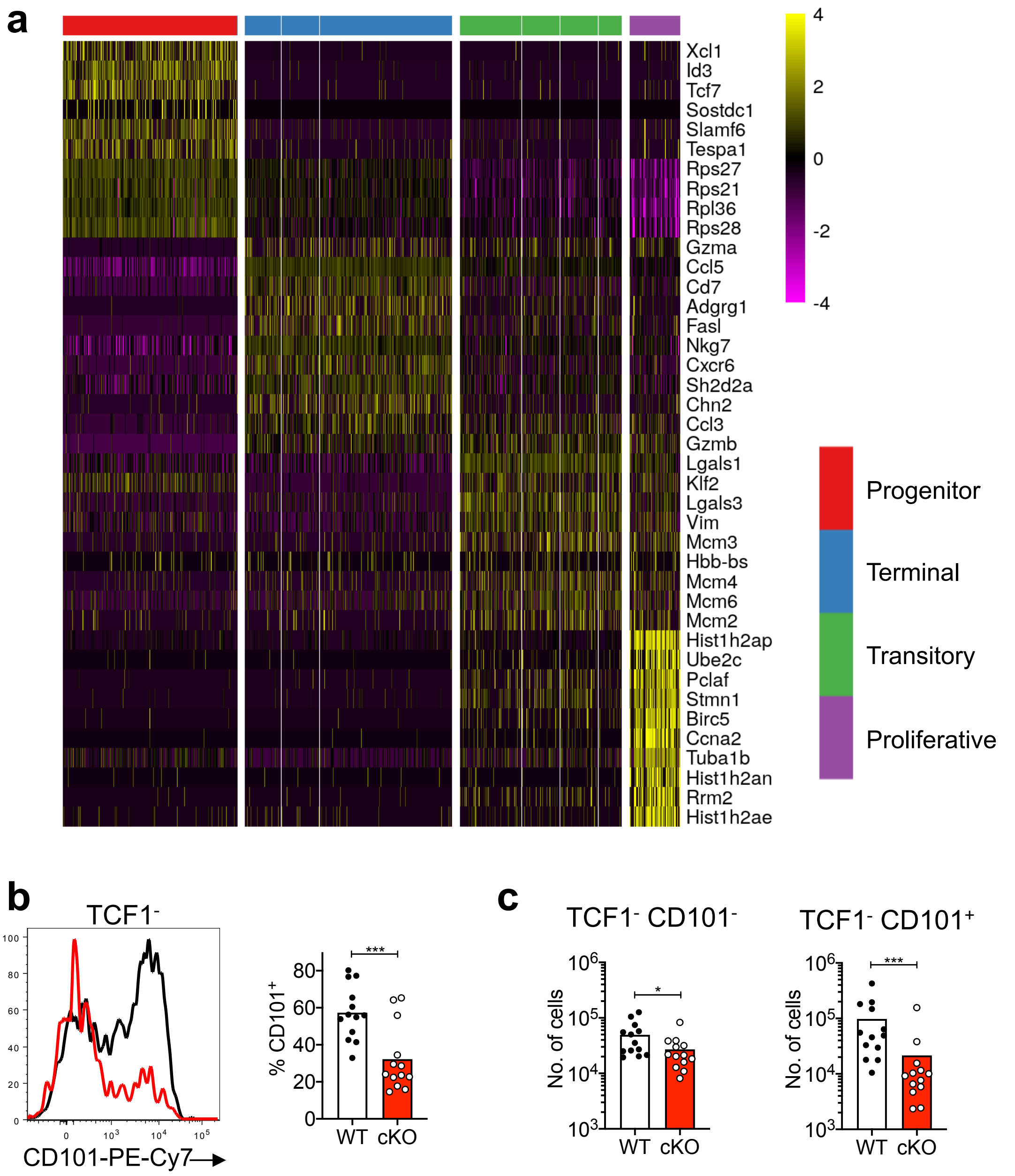

Supplementary Figure 5. Cell population analysis and validation of scRNAseq exhausted CD8$^+$ T cell clusters. (a) Z-score heatmap illustrating marker gene expression within the exhausted CD8$^+$ T cell clusters identified by scRNAseq in Fig. 5a. **(b,c)** The percentage **(b)** and number **(c)** of TCF1$^-$CD101$^+$ and TCF1$^-$CD101$^-$ splenic H2-D^{b}GP$_{33-41}$ tetramer stained CD8$^+$ T cells at day 20 p.i. with LCMV-Cl13 in CD4-depleted WT or cKO mice. n=13 mice per group from 4 independent experiments. p-values were calculated using a two-tailed Mann-Whitney test (c) or a two-tailed unpaired T test (b). For indicated significant differences, exact p values for (b) = 0.0006, and (c) (left to right) = 0.0387, 0.0002. * = $p<0.05$, *** = $p<0.001$.

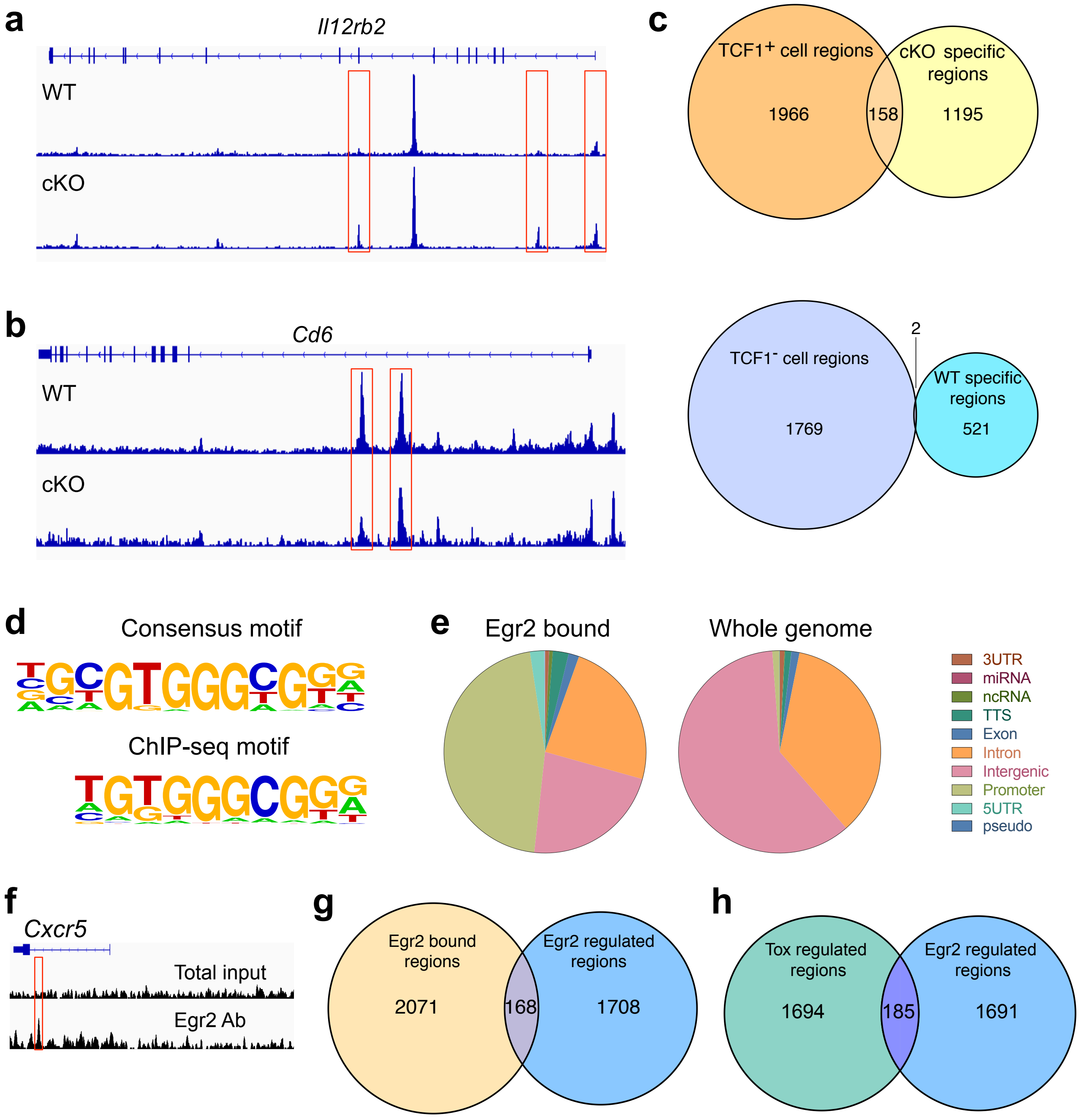
a
Il12rb2
WT
cKO
b
Cd6
WT
cKO
c
TCF1+ cell regions
1966
158
cKO specific regions
1195
TCF1- cell regions
1769
2
WT specific regions
521
d
Consensus motif
ChIP-seq motif
e
Egr2 bound
Whole genome
3UTR
miRNA
ncRNA
TTS
Exon
Intron
Intergenic
Promoter
5UTR
pseudo
f
Cxcr5
Total input
Egr2 Ab
g
Egr2 bound regions
2071
168
Egr2 regulated regions
1708
h
Tox regulated regions
1694
185
Egr2 regulated regions
1691

Supplementary Figure 6. ATACseq and EGR2 ChIP-seq analysis of CD8$^+$ T cells isolated from LCMV-Cl13 infection. **(a,b)** Representative ATACseq traces from the experiment in Fig. 6 of increased (*Il12rb*) **(a)** and decreased (*Cd6*) **(b)** accessibility in cKO cells at genes identified by RNAseq from the experiment in Fig. 3 as up- or down-regulated respectively in cKO cells. Red boxes indicate DORs. **(c)** Overlap between EGR2 cKO (top) or WT (bottom) specific regions, and published differentially accessible regions in TCF1$^+$ or TCF1$^-$ cells respectively. **(d-f)** CD8$^+$ T cells were isolated from LCMV-Cl13 infected B6 mice at day 20 p.i. and subjected to EGR2 ChIP-seq analysis. **(d)** Consensus EGR2 motif within the HOMER database (top), and the *de novo* EGR2 motif extracted from the ChIP-seq peaks (bottom). **(e)** Proportions of EGR2 binding within different genomic regions (left) relative to the proportion of those regions within the whole genome (right). **(f)** EGR2 ChIP-seq peak within the *Cxcr5* gene, which is induced by EGR2. Traces show EGR2 Ab signal, and Total input signal. **(g)** Overlap between EGR2 regulated regions from the ATACseq experiment in Fig. 6, and EGR2 ChIP-seq binding peaks. **(h)** Overlap between EGR2 regulated regions from the ATACseq experiment in Fig. 6, and published TOX regulated regions.

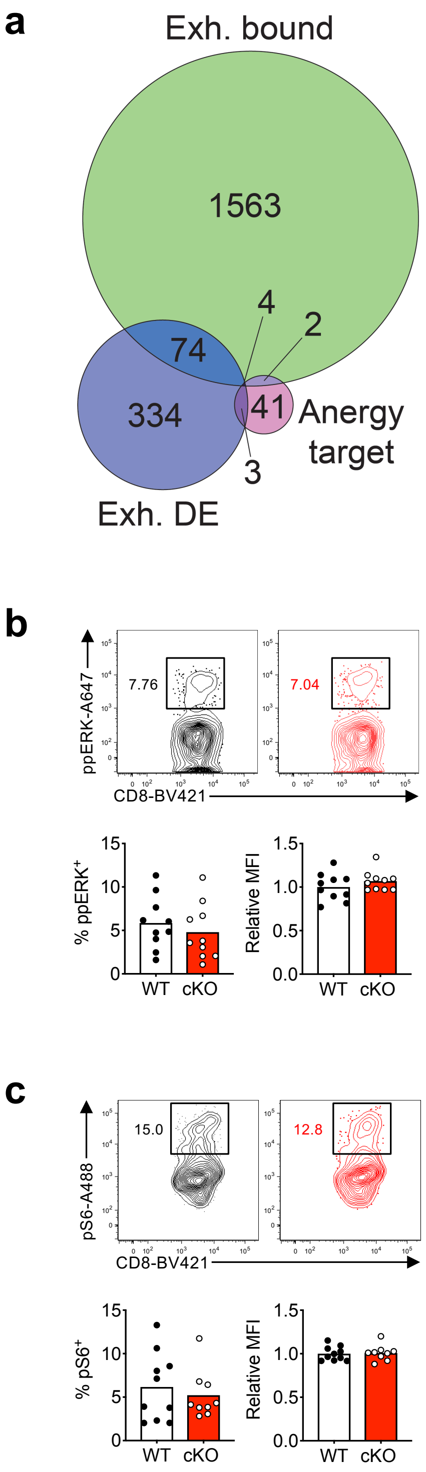

Supplementary Figure 7. The EGR2 exhaustion gene program is distinct from the EGR2 anergy program. (a) Venn diagram summarizing the overlap between genes identified as EGR2 bound by ChIP-seq within CD8^{+} T cells isolated from LCMV-Cl13 infection from Fig. 6 (Exh. bound), genes differentially expressed within exhausted cKO vs WT cells from Fig. 3c (Exh. DE), and genes previously identified as directly induced by EGR2 during anergy (Anergy target). **(b,c)** WT or cKO mice were depleted of CD4^{+} T cells and infected with LCMV-Cl13, then splenocytes were isolated at day 20 p.i. and restimulated with anti-CD3 for 30 min. CD44^{hi}PD-1^{hi} CD8^{+} T cells were then analysed for ppERK **(b)** and pS6 staining **(c)**. Relative MFI graphs depict MFI of the indicated marker within the marker positive population. n=9-10 mice per group from 2 independent experiments.

Supplementary Table 1 – Differentially expressed genes associated with differentially accessible chromatin regions in EGR2 cKO vs WT exhausted CD8^{+} T cells

Gene	Change in KO vs WT accessibility	Change in KO vs WT expression
Ifitm1	Increased	Up-regulated
Nav1	Increased	Up-regulated
Dnm3	Increased	Up-regulated
Fnip2	Increased	Up-regulated
Fbn1	Increased	Up-regulated
Il12rb2	Increased	Up-regulated
Fmnl2	Increased	Up-regulated
Gpr55	Increased	Up-regulated
Nav2	Increased	Up-regulated
Plcb4	Increased	Up-regulated
Specc1	Increased	Up-regulated
Itga1	Increased	Up-regulated
Pag1	Increased	Up-regulated
Adgrv1	Increased	Up-regulated
Nhsl2	Increased	Up-regulated
Trio	Increased	Up-regulated
Itsn1	Increased	Up-regulated
Satb1	Increased	Up-regulated
Il18r1	Increased	Up-regulated
Dst	Increased	Up-regulated
Kctd12	Increased	Up-regulated
Med12l	Increased	Up-regulated
Slco3a1	Increased	Up-regulated
Tanc1	Increased	Up-regulated
Diaph2	Increased	Up-regulated
Tiam1	Increased	Up-regulated
Iqgap2	Increased	Up-regulated
D1Ertd622e	Increased	Up-regulated
Parp8	Increased	Up-regulated
Plxnc1	Increased	Up-regulated
Runx2	Increased	Up-regulated
Il18rap	Increased	Up-regulated
Prrc2c	Increased	Up-regulated
Klrd1	Increased	Up-regulated
Slc7a1	Increased	Up-regulated
Itpr1	Increased	Up-regulated
Klrc2	Increased	Up-regulated
Cit	Increased	Up-regulated
Dnajc15	Increased	Up-regulated
Klrk1	Increased	Up-regulated
Notch4	Decreased	Up-regulated

Aif1	Decreased	Up-regulated
Itgad	Decreased	Up-regulated
Myo9a	Decreased	Up-regulated
Plcg2	Decreased	Up-regulated
Plekhm3	Decreased	Up-regulated
Aldh2	Decreased	Up-regulated
Sh2b3	Decreased	Up-regulated
Kdm6b	Decreased	Up-regulated
Tmem131	Decreased	Up-regulated
Zbtb37	Decreased	Up-regulated
Ppp1r15a	Decreased	Up-regulated
Kmt2a	Decreased	Up-regulated
Ubr4	Decreased	Up-regulated
Srrm2	Decreased	Up-regulated
Cad	Decreased	Up-regulated
Smpdl3a	Increased	Down-regulated
Lrig1	Increased	Down-regulated
Galm	Increased	Down-regulated
Itga9	Increased	Down-regulated
Gpr65	Decreased	Down-regulated
Gm20388	Decreased	Down-regulated
Smpdl3b	Decreased	Down-regulated
Cd5	Decreased	Down-regulated
Abcb9	Decreased	Down-regulated
Cd6	Decreased	Down-regulated
Mien1	Decreased	Down-regulated

Supplementary Table 2 – HOMER *de novo* transcription factor binding motifs identified within EGR2 ChIP-seq binding peaks. p values were calculated using HOMER by enrichment tests using a cumulative binomial distribution.

P-value	% of Targets	% of Background	Best Match
1.00E-206	38.77%	11.80%	Etv2(ETS)
1.00E-92	17.21%	4.73%	RUNX1(Runt)
**1.00E-65**	**31.45%**	**15.87%**	**Egr2(Zf)**
1.00E-43	16.20%	6.97%	Atf1(bZIP)
1.00E-32	0.86%	0.01%	PB0099.1_Zfp691_1
1.00E-30	0.81%	0.01%	MF0009.1_TRP(MYB)_class
1.00E-30	0.81%	0.01%	ZBTB7B
1.00E-29	6.87%	2.20%	IRF2(IRF)
1.00E-28	1.11%	0.03%	KLF10(Zf)
1.00E-27	0.76%	0.01%	PB0117.1_Eomes_2
1.00E-27	4.39%	1.04%	GFX(?)
1.00E-25	0.76%	0.01%	PB0056.1_Rfxdc2_1
1.00E-22	0.56%	0.00%	HINFP
1.00E-21	18.02%	10.71%	NRF(NRF)
1.00E-20	0.50%	0.00%	GCM2
1.00E-20	0.50%	0.00%	CEBPA
1.00E-20	0.50%	0.00%	SP4
1.00E-19	0.56%	0.01%	PB0091.1_Zbtb3_1
1.00E-18	5.65%	2.14%	FOSL1
1.00E-17	0.45%	0.00%	Maz(Zf)
1.00E-17	7.47%	3.40%	NFY(CCAAT)
1.00E-17	0.71%	0.02%	EKLF(Zf)
1.00E-17	0.76%	0.03%	PB0022.1_Gata5_1
1.00E-16	8.68%	4.35%	Mef2c(MADS)
1.00E-15	6.51%	2.93%	NFY(CCAAT)
1.00E-15	0.40%	0.00%	CRX(Homeobox)
1.00E-15	0.81%	0.04%	PB0196.1_Zbtb7b_2
1.00E-14	5.50%	2.39%	NFAT(RHD)
1.00E-13	0.45%	0.01%	PH0017.1_Cux1_2
1.00E-10	4.09%	1.78%	PB0176.1_Sox5_2
1.00E-09	0.30%	0.01%	SPI1
1.00E-08	0.76%	0.10%	PB0179.1_Sp100_2
1.00E-05	4.90%	2.97%	YY2/MA0748.1
1.00E-03	1.41%	0.71%	Ap4(bHLH)

Supplementary Table 3 – Directly bound EGR2 targets during $CD8^{+}$ T cell exhaustion

Gene	Up or down in KO during exhaustion?	EGR2 anergy target?
Nr4a3	Up	No
Cd74	Up	Yes (down in KO in anergy)
Cst3	Up	No
Il18r1	Up	No
Satb1	Up	No
Cbfa2t3	Up	No
Ly6c2	Up	No
Irf8	Up	No
Bach2	Up	Yes (down in KO in anergy)
Il12rb2	Up	No
Ryr1	Up	Yes (down in KO in anergy)
Rab43	Up	No
Kmt2d	Up	No
Zeb2	Up	No
Ahnak	Up	No
Jun	Up	No
Hivep3	Up	No
D1Ertd622e	Up	No
Nr4a1	Up	No
Rara	Up	No
Utrn	Up	No
Kdm7a	Up	No
Kmt2a	Up	No
Gvin1	Up	No
Gm4070	Up	No
Bcl6	Up	No
Ubr4	Up	No
Ssh2	Up	No
Egr3	Up	No
Parp8	Up	No
Srrm2	Up	No
Btla	Up	No
Sh2b3	Up	No
Iqgap2	Up	No
Diaph2	Up	No
Zfp36l1	Up	No
Itpr1	Up	No
Smad3	Up	No
Dnah6	Up	No
Plec	Up	No
Mtmr4	Up	No
Birc6	Up	No

Marcks	Up	No
Fbxw7	Up	No
Mycbp2	Up	No
Luc7l2	Up	No
Atrn	Up	No
Klrk1	Up	No
Med12l	Up	No
Ppp1r15a	Up	No
Tox2	Down	No
Egr2	Down*	Yes (down in KO in anergy)*
Izumo1r	Down	No
Abcb9	Down	No
Tigit	Down	No
Pdcd1	Down	No
Ephx1	Down	No
Rgs1	Down	No
Prr7	Down	No
Ift27	Down	No
Gimap7	Down	No
Hist1h4i	Down	No
Galnt2	Down	No
Hist1h4k	Down	No
Fasl	Down	No
Nab1	Down	No
Prkch	Down	No
Hist1h2bf	Down	No
Ctsc	Down	No
Gpr65	Down	No
Glipr1	Down	No
Cd3d	Down	No
Adap1	Down	No
Sub1	Down	No
Hist1h2be	Down	No
Chn2	Down	No
Cxcr5	Down	No
Hist1h2br	Down	No

* In both datasets *Egr2* was knocked out, meaning that *Egr2* expression was reduced in KO cells in both datasets due to gene deletion rather than differential expression

Supplementary Table 4 – Differentially enriched HOMER motifs within EGR2 cKO vs WT-specific ATAC-seq peaks

Best match	Log2(fold enrichment)	Difference in p-value z-score (absolute value)
WRKY21	2.6138595	3.0902323
Fosl2	1.9941754	3.7190165
Jun-AP1	1.6492643	3.7190165
JunB	1.2621881	4.2648908
Fra2	1.1573544	3.7190165
AP-1	1.1017486	3.7190165
Fra1	0.9777828	4.2648908
Atf3	0.8995808	3.0902323
ATHB53	0.8339160	3.0902323
BATF	0.7742913	3.7190165
TCP20	-0.7149613	3.7190165
TR4	-0.7190281	3.0902323
E2FA	-0.7266937	3.0902323
THRb	-0.7463093	3.0902323
MYB113	-0.7544676	3.0902323
ANAC075	-0.7547403	3.0902323
ANAC005	-0.7565208	4.2648908
MYB116	-0.7586791	5.1993376
p63	-0.7636947	3.0902323
TCP7	-0.7678516	3.7190165
Bcl11a	-0.7921711	3.0902323
FOXK2	-0.8277438	4.2648908
HINFP	-0.8669952	3.7190165
RBPJ:Ebox	-0.8882749	3.0902323
At4g18890	-0.8948437	3.0902323
Egr1	-0.9024209	3.7190165
CELF2	-0.9161363	3.0902323
AT1G24250	-0.9176127	4.2648908
VDR	-0.9507219	3.0902323
DPL-1	-1.0012826	3.7190165
PAX6	-1.0740913	3.0902323
NRF	-1.1721635	6.7060232
SPL5	-1.4421104	3.0902323
bHLH28	-1.8054529	3.0902323
HSF21	-1.8856251	3.0902323
TCP1	-2.0307854	4.2648908
NRF1	-2.2837969	6.3613409
YY1	-3.6200478	4.7534243

Red indicates AP-1 family motif

Supplementary Table 5 – Buffers for ChIP-seq analysis

Buffer Name	Recipe
10X Formaldehyde Cross-Linking Buffer	50mM HEPES-KOH (pH 7.5)<br>100mM NaCl<br>1mM EDTA<br>11% Formaldehyde
Nuclear Extraction Buffer	20mM Tris-HCl (pH 8.0)<br>10mM NaCl<br>2mM EDTA<br>0.5% IGEPAL CA-630
ChIP Sonication Buffer	20mM Tris-HCl (pH 7.5)<br>150mM NaCl<br>2mM EDTA<br>1% IGEPAL CA-630<br>0.3% SDS
ChIP Dilution Buffer	20mM Tris-HCl pH 8.0<br>150mM NaCl<br>2mM EDTA<br>1% TritonX-100
ChIP IP Buffer	20mM Tris-HCl (pH 8.0)<br>150M NaCl<br>2mM EDTA<br>1% TritonX-100<br>0.15% SDS
ChIP Blocking Buffer	ChIP IP Buffer<br>0.1% BSA
ChIP Wash Buffer 1	20mM Tris-HCl pH 8.0<br>500mM NaCl<br>2mM EDTA<br>1% TritonX-100<br>0.1% SDS
ChIP Wash Buffer 2	20mM Tris-HCl pH 8.0<br>250mM LiCl<br>2mM EDTA<br>0.5% IGEPAL CA-630<br>0.1% SDS<br>0.5% Sodium Deoxycholate
Reverse Crosslinking Buffer	1% SDS<br>100mM $NaHCO_3$<br>200mM NaCl
