# Supplementary material for: Antigen-driven EGR2 expression is required for exhausted CD8+ T cell stability and maintenance
Source: Nat Commun. 2021 May 13;12:2782. doi: 10.1038/s41467-021-23044-9 (PMC8119420; doi:10.1038/s41467-021-23044-9)
Supplement: Supplementary file 2 — Description of Additional Supplementary Files [file 41467_2021_23044_MOESM2_ESM.pdf]

Description of Additional Supplementary Files

Supplementary Data 1 – significantly differentially expressed genes within GFP+ vs GFP- polyclonal CD8+CD44int-hiPD-1+Slamf6+Tim3- cells (FDR<0.05). p values were calculated using a moderated T test in Limma with a two-sided analysis and a Benjamini-Hochberg multiple comparison adjustment.

Supplementary Data 2 – significantly differentially expressed genes within tetramer+ Egr2 cKO CD8+ T cells relative to WT control cells (FDR<0.05). p values were calculated using a moderated T test in Limma with a two-sided analysis and a Benjamini-Hochberg multiple comparison adjustment.

Supplementary Data 3 – significantly differentially expressed genes within cKO vs WT scRNAseq exhausted CD8+ T cell clusters (FDR<0.05, log2 fold change >0.8 or <-0.8). p values were calculated using the quasi-likelihood F-test implemented in EdgeR with a twosided analysis and a Benjamini-Hochberg multiple comparison adjustment.

Supplementary Data 4 – significantly differentially open chromatin regions within tetramer+ Egr2 cKO CD8+ T cells relative to WT control cells (FDR<0.01), including overlap with TOX-regulated regions and EGR2 binding sites. p values were calculated using the quasi-likelihood F-test implemented in csaw with a two-sided analysis and a Benjamini-Hochberg multiple comparison adjustment.

Supplementary Data 5 – AP-1 transcription factor expression in exhausted cell scRNAseq clusters. p values were calculated using the quasi-likelihood F-test implemented in EdgeR with a two-sided analysis and a Benjamini-Hochberg multiple comparison adjustment.
