# Supplementary material for: Antigen-driven EGR2 expression is required for exhausted CD8+ T cell stability and maintenance
Source: Nat Commun. 2021 May 13;12:2782. doi: 10.1038/s41467-021-23044-9 (PMC8119420; doi:10.1038/s41467-021-23044-9)
Supplement: Supplementary file 8 — Reporting Summary [file 41467_2021_23044_MOESM8_ESM.pdf]

nature research

Corresponding author(s): Ian Parish, Christopher Goodnow

Last updated by author(s): Mar 3, 2021

Reporting Summary

Nature Research wishes to improve the reproducibility of the work that we publish. This form provides structure for consistency and transparency in reporting. For further information on Nature Research policies, see our Editorial Policies and the Editorial Policy Checklist.

Statistics

For all statistical analyses, confirm that the following items are present in the figure legend, table legend, main text, or Methods section.

n/a	Confirmed	
☐	☒	The exact sample size (*n*) for each experimental group/condition, given as a discrete number and unit of measurement
☐	☒	A statement on whether measurements were taken from distinct samples or whether the same sample was measured repeatedly
☐	☒	The statistical test(s) used AND whether they are one- or two-sided<br>*Only common tests should be described solely by name; describe more complex techniques in the Methods section.*
☒	☐	A description of all covariates tested
☐	☒	A description of any assumptions or corrections, such as tests of normality and adjustment for multiple comparisons
☐	☒	A full description of the statistical parameters including central tendency (e.g. means) or other basic estimates (e.g. regression coefficient) AND variation (e.g. standard deviation) or associated estimates of uncertainty (e.g. confidence intervals)
☐	☒	For null hypothesis testing, the test statistic (e.g. *F*, *t*, *r*) with confidence intervals, effect sizes, degrees of freedom and *P* value noted<br>*Give P values as exact values whenever suitable.*
☒	☐	For Bayesian analysis, information on the choice of priors and Markov chain Monte Carlo settings
☒	☐	For hierarchical and complex designs, identification of the appropriate level for tests and full reporting of outcomes
☒	☐	Estimates of effect sizes (e.g. Cohen's *d*, Pearson's *r*), indicating how they were calculated

Our web collection on statistics for biologists contains articles on many of the points above.

Software and code

Policy information about availability of computer code

Data collection	Flow cytometry data was collected on the flow cytometers listed in the methods using FACSDiva software (BD Pharmingen, v8.0.1).
Data analysis	Flow cytometry data was analysed using FlowJo software (Treestar, v9.9.6), data calculations were conducted in Microsoft Excel (v14.7.2) while data was graphed and statistical analysis conducted using Prism (GraphPad, v8.1.2). For bioinformatic analysis the following software was used: HISAT2 (v2.1.0), FeatureCounts from the Subread package (v1.5.0-p3), LIMMA (v3.32.4), Pheatmap (v1.0.8), ggplot2 (v2.2.1), GSEA desktop application (v3.0 beta 1), bcl2fastq (v2.17.1.14), bowtie2 (v2.3.3), Samtools (v1.4.1), MACS (v2.1.1), Bedtools (v2.26), Homer (v4.8), IGVTools (v2.3.95), IGV (v2.3.55), Trim_Galore (v0.4.5_dev), BWA-MEM (v0.7.17), MarkDuplicates (Picard) (v2.19.0), ATACseqQC (v1.10.1), csaw (v1.20.0), Cell Ranger v3.1.0, EdgeR (v3.28.0).

For manuscripts utilizing custom algorithms or software that are central to the research but not yet described in published literature, software must be made available to editors and reviewers. We strongly encourage code deposition in a community repository (e.g. GitHub). See the Nature Research guidelines for submitting code & software for further information.

Data

Policy information about availability of data

All manuscripts must include a data availability statement. This statement should provide the following information, where applicable:

- Accession codes, unique identifiers, or web links for publicly available datasets
- A list of figures that have associated raw data
- A description of any restrictions on data availability

The raw sequencing data associated with Fig. 3, 5 and 6, and Supplementary Fig. 1, is available in the NCBI GEO repository at accession number GSE134710. The gene lists used for GSEA analysis in Supplementary Fig. 1d were extracted from Table S1 in the cited Beltra et al study (available at https://doi.org/10.1016/j.immuni.2020.04.014). The gene lists used for GSEA analysis in Figure 3 were taken from the publicly available dataset GSE84105 within the NCBI GEO database. The

gene lists used for GSEA in Fig. 5 were extracted from supplementary data in the cited Man et al study (available at https://doi.org/10.1016/j.immuni.2017.11.021). TOX DORs (Supplementary Fig. 6h) were obtained from Supplementary Table 5 of the cited Khan et al paper. TCF1+ and TCF1- cell-specific ATACseq peaks were obtained by reanalysis of sequencing data from the cited Jadhav et al paper from the publicly available dataset PRJNA546023 within the NCBI BioProject database. All sequencing data was mapped and annotated using reference genome GRCm38/mm10 (https://www.ncbi.nlm.nih.gov/assembly/GCF_000001635.20/) and Ensembl gene annotation release 93 (http://ftp.ensembl.org/pub/release-93/).

Field-specific reporting

Please select the one below that is the best fit for your research. If you are not sure, read the appropriate sections before making your selection.

- [x] Life sciences
- [ ] Behavioural & social sciences
- [ ] Ecological, evolutionary & environmental sciences

For a reference copy of the document with all sections, see nature.com/documents/nr-reporting-summary-flat.pdf

Life sciences study design

All studies must disclose on these points even when the disclosure is negative.

Sample size	Sample sizes were chosen in mouse experiments based on prior experience of the "n" required to achieve significance given the known experimental variation from our previous work (eg. Parish et al JCI 2014).
Data exclusions	No data was excluded.
Replication	All reported experiments were conducted at least two times, and all findings reported were successfully reproduced. The only exception was the ChIP-seq experiment, which was performed once but on cells pooled from 8 mice. However, we were able to successfully independently validate the peaks by demonstrating that as expected EGR2 ChIP-seq signal strength correlated with chromatin accessibility from an independently generated ATAC-seq dataset, and by demonstrating that the EGR2 binding motif was significantly enriched within ChIP-seq peaks (Supplementary Table 2, and Supplementary Figure 6d). The scRNAseq experiment in Fig. 5 was also performed once on pooled cells from 3 mice per genotype, however all major findings from the scRNAseq analysis were successfully replicated multiple times both by FACS analysis and/or the bulk RNAseq study.
Randomization	In experiments where there were different treatment conditions (eg. adoptive transfer experiments, chronic vs acute infection comparisons etc), mice were randomly assigned to groups, and age and sex matched mice were used. Littermate controls were used in genetically modified strains, with all mice of a similar age and/or sex where possible.
Blinding	While blinding was not always feasible due to cage labelling and animal housing requirements, where possible, downstream analysis of samples (ie. sample processing, flow cytometric analysis) was conducted in a manner blinded to mouse genotype. In particular, tumour growth studies were conducted blinded to genotype.

Reporting for specific materials, systems and methods

We require information from authors about some types of materials, experimental systems and methods used in many studies. Here, indicate whether each material, system or method listed is relevant to your study. If you are not sure if a list item applies to your research, read the appropriate section before selecting a response.

Materials & experimental systems

n/a	Involved in the study	
[ ]	[x]	Antibodies
[ ]	[x]	Eukaryotic cell lines
[x]	[ ]	Palaeontology and archaeology
[ ]	[x]	Animals and other organisms
[x]	[ ]	Human research participants
[x]	[ ]	Clinical data
[x]	[ ]	Dual use research of concern

Methods

n/a	Involved in the study	
[ ]	[x]	ChIP-seq
[ ]	[x]	Flow cytometry
[x]	[ ]	MRI-based neuroimaging

Antibodies

Antibodies used	Antibodies are listed below with target, clone, supplier (Biolegend unless otherwise stated), dilution and catalog number: CD8a (clone 53-6.7; PerCP (1/100, Cat. no. 100732), BV421 (1/200, Cat. no. 100738), BUV395 (1/200, BD Biosciences Cat. no. 563786) and BUV805 (1/200, BD Biosciences Cat. no. 563786)), CD44 (clone IM7; Pacific Blue (1/400, Cat. no. 103020), BUV737 (1/1000, BD Biosciences Cat. no. 612799)), CD107a (clone 1D4B; FITC (1/400, Cat. no. 121606)), IFN (clone XMG1.2; PE-Cy7 (1/2000, eBioscience Cat. no. 25-7311-41), TNFa (clone MP6-XT22; PE (1/2000, Cat. no. 506306)), IL-2 (clone JES6-5H4; APC (1/100, eBioscience Cat. no. 17-7021-82)), CD45.1 (clone A20; FITC (1/200, BD Biosciences Cat. no. 553775)), CD45.2 (clone 104; BUV395 (1/200, BD Biosciences Cat. no. 564616)), Ly6C (clone AL21; FITC (1/200, BD Biosciences Cat. no. 553104)), PD-1 (clone 29F.1A12; BV785 (1/100, Cat. no. 135225)), Tim3 (clone RMT3-23; BV605 (1/100, Cat. no. 119721)), 2B4 (clone 2B4; FITC (1/50, BD Biosciences Cat. no. 553305)), Lag3 (clone C9B7W; PE (1/100, Cat. no. 125208)), CD160 (clone 7H1; PE-Cy7 (1/100, Cat. no. 143010)), Egr2 (clone

	erongr2; PE (1/50, eBioscience Cat. no. 143010)), TCF1 (clone C63D9; unconjugated (1/100, Cell Signaling Technologies Cat. no. 2203S) followed by a secondary antibody (Goat anti-Rabbit Alexa594 (1/1000, Invitrogen Cat. no. A11012))), CXCR5 (clone 2G8; BV421 (1/25, BD Biosciences Cat. no. 562856)), Slamf6 (clone 13G3; PE (1/200, BD Biosciences Cat. no. 561540), BV421 (1/200, BD Biosciences Cat. no. 740090)), CD101 (clone Moushi101; PE-Cy7 (1/200, eBioscience Cat. no. 25-1011-82)), CD127 (clone A7R34; PE-Cy7 (1/100, Cat. no. 135014)), TIGIT (clone 1G9; BV650 (1/100, BD Biosciences Cat. no. 744213)), Ly6A/E (clone E13-161.7; PE (1/200, BD Biosciences Cat. no. 553336)), GzmB (clone GB11; PE (1/200, eBioscience Cat. no. GRB04)), FR4 (clone eBio12A5; PE-Cy7 (1/100, eBioscience Cat. no. 25-5445-82)), IL-10Ra (clone 1B1.3a; PE (1/100, Cat. no. 112706)), KLRG1 (clone 2F1; FITC (1/200, eBioscience Cat. no. 11-5893-82)), CD62L (clone MEL-14; APC-Cy7 (1/200, Cat. no. 104428)), Eomes (clone Dan11mag; Alexa488 (1/50, eBioscience Cat. no. 53-4875-82)), ppErk1/2 (clone 197G2; Alexa647 (1/100, Cell Signaling Technologies Cat. no. 13148S)), and pS6 (clone D57.2.2E; Alexa488 (1/100, Cell Signaling Technologies Cat. no. 4803S)).
Validation	The Egr2 antibody staining seen within virus-specific CD8+ T cells in LCMV Clone 13 infection was validated by comparing to background staining in Egr2 KO cells. Representative staining is shown for a number of antibodies within Figures 1, 2, 3, 4, 5 and Supplementary Figures 1, 2, 3, 5, 7. Further validation data is provided on the manufacturer's website, which illustrates target staining relative to isotype control antibodies. Validation data can be accessed by searching for the listed catalog numbers on the Biolegend (https://www.biolegend.com/), eBioscience/Invitrogen/Thermofisher (https://www.thermofisher.com/), BD Biosciences (https://www.bdbiosciences.com/) or Cell Signaling Technology (https://www.cellsignal.com/) websites.

Eukaryotic cell lines

Policy information about cell lines

Cell line source(s)	B16-OVA cells were a gift from J. Oliaro, and the parental B16-F10 line was originally obtained from ATCC.
Authentication	B16 cells were not authenticated by genetic profiling.
Mycoplasma contamination	All cells used routinely tested negative for mycoplasma.
Commonly misidentified lines (See ICLAC register)	No commonly misidentified cell lines were used in this study.

Animals and other organisms

Policy information about studies involving animals; ARRIVE guidelines recommended for reporting animal research

Laboratory animals	All described mouse strains were on a C57BL/6J background. Both male and female mice were used, with mice typically used for experiments at 8-12 weeks of age. All mice were housed in IVC caging, supplied with irradiated shredded corn cob bedding and irradiated mouse feed diet. The light cycle was 14 hours light and 10 hours dark with a half our sunrise and sunset phase programmed into the cycle. The ambient temperature of each room was set at 20oC +/- 1oC. The temperature inside the boxes generally sat between 22 and 24oC. Humidity was set between 40 to 60%.
Wild animals	The study did not involve wild animals.
Field-collected samples	The study did not involve samples collected from the field.
Ethics oversight	All animal work was in accordance with protocols approved by the ANU and Peter MacCallum Cancer Centre Animal Experimentation Ethics Committees, and current guidelines from the Australian Code of Practice for the Care and Use of Animals for Scientific Purposes.

Note that full information on the approval of the study protocol must also be provided in the manuscript.

ChIP-seq

Data deposition

- [x] Confirm that both raw and final processed data have been deposited in a public database such as GEO.
- [x] Confirm that you have deposited or provided access to graph files (e.g. BED files) for the called peaks.

Data access links *May remain private before publication.*	https://www.ncbi.nlm.nih.gov/geo/query/acc.cgi?acc=GSE134710
Files in database submission	RNAseq (2x Egr2 WT, 2x Egr2 KO), RNAseq (2x Egr2-GFP+, 2x Egr2-GFP-), ChIP-seq (1x EGR2-ChIP, 1x input, 1x BED file), ATACseq (2x Egr2 WT, 2x Egr2 KO), scRNAseq (cell transcriptome data file, cell hashtag data file)
Genome browser session (e.g. UCSC)	No longer applicable.

Methodology

Replicates	The ChIP-seq analysis was conducted as a single replicate using CD8+ T cells pooled from 8 mice, with both EGR2 antibody and total input control samples generated.
Sequencing depth	Libraries were pooled and sequenced with 75 bp single-end sequencing to a depth of 15–20x10^6 reads.

Antibodies	A previously validated polyclonal anti-EGR2 ChIP antibody (ab43020, Abcam) was used for ChIP-seq analysis.
Peak calling parameters	Mapping was performed with Bowtie2(v2.3.3) with default options. Resulting sam files were converted to Bams, sorted, and duplicates removed using Samtools (v1.4.1). macs2 (v2.1.1) was used for peak calling using default parameters. An input sample was used as a control for peak calling.
Data quality	Of the 2258 peaks identified as enriched over input, there were 1378 peaks with a q<0.05 and a >5 fold enrichment.
Software	bcl2fastq (v2.17.1.14) was used for de-multiplexing. The Fastq files were then aligned to the mouse reference genome (GRCm38/mm10) using bowtie2 (v2.3.3). Samtools (v1.4.1) was used for manipulation of SAM and BAM files. MACS (v2.1.1) was used for peak-calling, and significant peaks (FDR<0.05) were then associated with the closest gene TSS using Bedtools (v2.26). Motif analysis was performed with Homer (v4.8). Browser viewable TDF files were generated using IGVTools (v2.3.95) and ChIP-Seq tracks were visualized using IGV (v2.3.55). Graphics were generated using deeptools (v2.5.3).

Flow Cytometry

Plots

Confirm that:

- [x] The axis labels state the marker and fluorochrome used (e.g. CD4-FITC).
- [x] The axis scales are clearly visible. Include numbers along axes only for bottom left plot of group (a 'group' is an analysis of identical markers).
- [x] All plots are contour plots with outliers or pseudocolor plots.
- [x] A numerical value for number of cells or percentage (with statistics) is provided.

Methodology

Sample preparation	Spleens were harvested from mice and processed into a single cell suspension by mashing through a 70µM filter. Samples were RBC lysed in 0.83% NH4Cl then filtered again prior to analysis. For TIL analysis, isolated tumours were digested on a gentleMACS Dissociator (Miltenyi Biotec) at day 14 post-inoculation in C Tubes (Miltenyi Biotec) using the mouse Tumour Dissociation Kit (Miltenyi Biotec) and the program "37C_m_TDK_1" according to manufacturer's instructions. Cells were then washed, resuspended in a 44% Percoll solution, underlaid with a 56% Percoll solution, then centrifuged on low brake. TILs were isolated from the interphase, RBC lysed in 0.83% NH4Cl, and then stained for flow cytometric analysis.
Instrument	BD LSRII, Fortessa, X20 or Symphony instruments were used for analysis. BD FACS Aria II was used for cell sorting.
Software	BD FACS Diva software (v8.0.1) was used to collect data, while FlowJo software (Treestar, v9.9.6) was used for data analysis.
Cell population abundance	Purity was >90% in all samples after sorting as assessed by reanalysis of the sample post-sort.
Gating strategy	Gating strategies are illustrated in Supplementary Fig. 1a (tetramer), 1e (TILs) and 2a (cytokine staining), with the flow cytometry data relevant to each gating strategy referenced in the figure legends. A broad FSC/SSC gate was used to eliminate small debris, doublets were excluded using FSC-H vs FSC-W, dead cells were then excluded using a fixable viability stain after which CD8+ cells were gated upon for subsequent analysis (either for gating on tetramer+ cells, PD-1+ cells or on IFNg+ cells). For TIL analysis, a DUMP gate containing CD19, NK1.1 and F4/80 antibodies was also included along with a CD45 antibody, and CD8+ cells were then additionally gated to ensure that they were DUMP-CD45+.

- [x] Tick this box to confirm that a figure exemplifying the gating strategy is provided in the Supplementary Information.
